# An improved synthesis of a fluorophosphonate–polyethylene glycol–biotin probe and its use against competitive substrates

**DOI:** 10.3762/bjoc.9.12

**Published:** 2013-01-15

**Authors:** Hao Xu, Hairat Sabit, Gordon L Amidon, H D Hollis Showalter

**Affiliations:** 1 Department of Medicinal Chemistry, University of Michigan, Ann Arbor, MI 48109-1065, USA; 2 Department of Pharmaceutical Sciences, University of Michigan, Ann Arbor, MI 48109-1065, USA; 3 Vahlteich Medicinal Chemistry Core, University of Michigan, Ann Arbor, MI 48109-1065, USA

**Keywords:** biotin, fluorophosphonate, high turnover rate, reversible substrate

## Abstract

The fluorophosphonate (FP) moiety attached to a biotin tag is a prototype chemical probe used to quantitatively analyze and enrich active serine hydrolases in complex proteomes in an approach called activity-based protein profiling (ABPP). In this study we have designed a novel synthetic route to a known FP probe linked by polyethylene glycol to a biotin tag (FP–PEG–biotin). Our route markedly increases the efficiency of the probe synthesis and overcomes several problems of a prior synthesis. As a proof of principle, FP–PEG–biotin was evaluated against isolated protein mixtures and different rat-tissue homogenates, showing its ability to specifically target serine hydrolases. We also assessed the ability of FP–PEG–biotin to compete with substrates that have high enzyme turnover rates. The reduced protein-band intensities resulting in these competition studies demonstrate a new application of FP-based probes seldom explored before.

## Introduction

One of the goals of chemical biology is to develop small–molecule- and biomolecule-based probes to interrogate biological processes. In this regard, fluorophosphonate (FP) probes have been extensively used in activity-based protein profiling (ABPP) in proteomic studies [1–2]. FP probes, specifically designed to target serine hydrolases, originate from diisopropyl fluorophosphonate (DFP) [3–4]. DFP is a serine hydrolase covalent inhibitor and from it have evolved analytical tools in which “handles“, such as biotin, rhodamine, and alkyne have been appended via a variety of linking chains [5–8]. These FP analogues have proven to be powerful tools in the profiling of complex proteome samples [9] and in the identification of selective inhibitors [10–11]. However, very few cases have been published in which these probes have been utilized in the study of enzymes with reversible substrates. These substrates are usually endogenous or exogenous organic molecules with small molecular weights. Their functional groups are enzymatically modified with high specificity and efficiency, behaving quite differently from inhibitors due to their high turnover rates. Thus, competition assays between substrates and FP probes are more difficult to perform due to the rapid kinetics and covalent binding properties of the probes.

**Figure 1:**
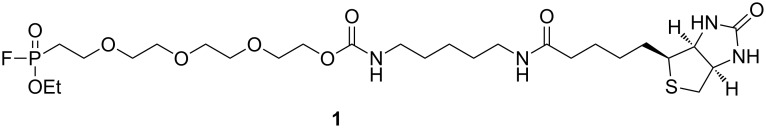
Structure of FP–PEG–biotin **1**.

The FP–PEG–biotin compound **1**, shown in Figure 1, was first synthesized by the Cravatt group and utilized for affinity isolation of enzymes by pull-down with avidin beads followed by mass-spectrometry analysis [6]. Our interest in generating **1** on a larger scale to use in our research led us to consider an alternative and more expeditious synthesis. This was driven by several concerns of the original route including (a) a poor overall yield, (b) challenges in the chromatographic purification of some intermediates due to their hydrophilicity and lack of a UV chromophore, and (c) generating and then carrying the fluorophosphonate moiety through two steps with an attendant concern of its high reactivity and potential for toxicity to the laboratory chemist [12–13]. In our efforts to address those shortcomings, we report a novel synthetic route to FP–PEG–biotin **1**, and provide some preliminary results of studies in which this probe was utilized in competition experiments with reversible enzyme substrates.

## Results

### Synthesis

**Scheme 1:**
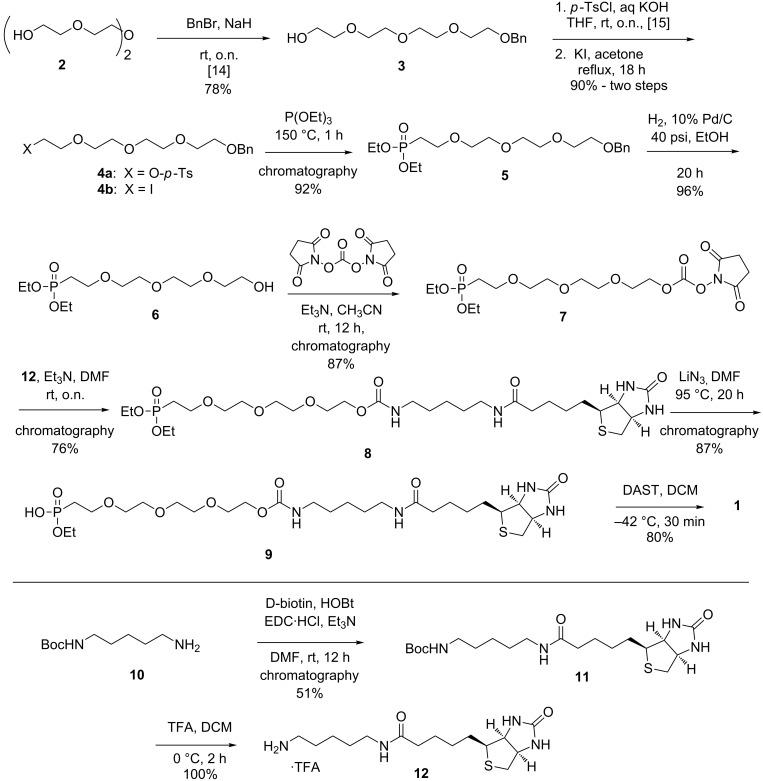
Synthetic scheme to FP–PEG–biotin probe **1**.

The route to target probe **1** is delineated in Scheme 1. Monobenzylation of tetraethylene glycol (**2**) was carried out by the procedure of Jiang et al. [14] to give ether **3** in 78% yield. This was then subjected to a two-step procedure to provide the novel iodo ether **4b**. Thus, tosylation of **3** utilizing a slight modification of literature conditions [15] gave **4a**, which was subjected to Finkelstein conditions by utilizing a procedure reported for a related compound [16] to afford **4b** in a combined 90% yield. The phosphonate moiety was installed under standard Arbuzov conditions by heating **4b** under reflux in neat triethyl phosphite for 1 h to provide novel intermediate **5** in 92% yield following purification by column chromatography. Clean removal of the benzyl protecting group under standard conditions of catalytic hydrogenolysis provided, in 96% yield, the known phosphonate polyether alcohol **6**, the synthesis of which had been accomplished previously by a different route [17]. Activation of the alcohol moiety of **6** to the succinimidyl carbonate **7** was carried out under standard conditions in 87% yield following chromatographic purification. Coupling of **7** to the in situ generated 5-(biotinamido)pentaneamine fragment **12**, made from Boc-protected diamine **10** in a known two-step process [18], was carried out under mild conditions to provide novel precursor **8** in 76% yield following purification. The stage was now set for a two-step modification of the phosphonate moiety. Reaction of **8** with lithium azide in hot DMF, under conditions developed for the monodealkylation of phosphonic acid dialkyl esters of nucleosides [19], provided the novel monoethyl ester **9** in 87% yield following purification by a two-stage chromatographic procedure. Penultimate intermediate **9** was then cleanly converted to the fluorophosphonate utilizing the standard fluoridating reagent (DAST in dichloromethane) at −42 °C. Workup provided the FP probe **1** [20] in 80% yield, which was pure enough to use directly in biological studies. Due to the absence of a UV chromophore, the purity of FP–PEG–biotin **1** could not be determined by HPLC. TLC analysis was also problematic due to its reactivity with the highly polar solvent mixture required to move it up a plate. Nevertheless, we deem **1** to be of high purity due to the cleanliness of its NMR spectra (^1^H, ^13^C, ^19^F, ^31^P). The structural assignments of all compounds were supported by diagnostic peaks in the ^1^H and ^13^C NMR spectra and by mass spectrometry. Digital copies of all NMR spectra are given in Supporting Information File 1.

### Evaluation of FP–PEG–biotin probe

**Figure 2:**
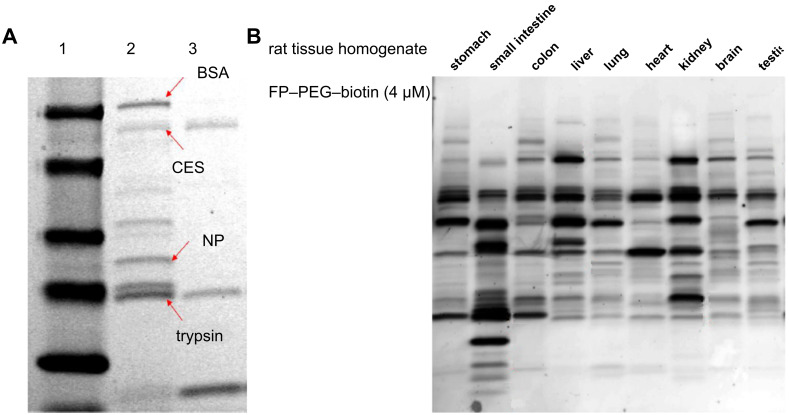
Labeling and affinity isolation of serine hydrolases by FP–PEG–biotin **1***.* (A) Lane 1: Protein standard. Lane 2: Mixture of BSA, pCES, NP, trypsin before purification. Lane 3: Purification results. (B) Rat tissue homogenates were prepared in 50 mM Tris buffer (pH 8.0) as 1 mg/mL samples from each tissue and incubated with FP–PEG–biotin **1** (4 μM) at room temperature for 1 h. Samples were quenched with 5× sample loading buffer, heated at 85 °C for 5 min, and separated by SDS-PAGE. Protein samples were transferred to a PVDF membrane, blotted by streptavidin alkaline phosphatase, and visualized by incubation with ECF substrate.

To validate the labelling efficiency of the FP–PEG–biotin probe **1** synthesized by our new route, we utilized it in the purification of four arbitrarily mixed proteins including bovine serum albumin (BSA), porcine carboxylesterase (pCES), nucleoside phosphorylase (NP), and trypsin (Figure 2A). Thus, the protein mixture was incubated with probe **1** for one hour followed by the addition of avidin agarose and further incubation. Trypsin and pCES, which are serine hydrolases, bound to the probe and were removed from the mixture, as shown in lane 3. We also assayed FP probe **1** against different rat-tissue samples as described in Supporting Information File 1. Rat stomach, small intestine, colon, liver, lung, heart, kidney, brain, testis proteome homogenates (1 mg/ml) in 50 mM Tris buffer (pH 8.0) were each treated with the FP probe for one hour at room temperature. Each run was separated on a gel and electrophoretically transferred onto a Hybond PVDF membrane and blotted with streptavidin. The results are shown in Figure 2B. The different patterns indicate that the FP probe can effectively select out serine hydrolase enzymes amongst the various tissue samples examined, as had been observed previously in studies by the Cravatt group [21].

### Kinetic study of FP probe labelling activity

**Figure 3:**
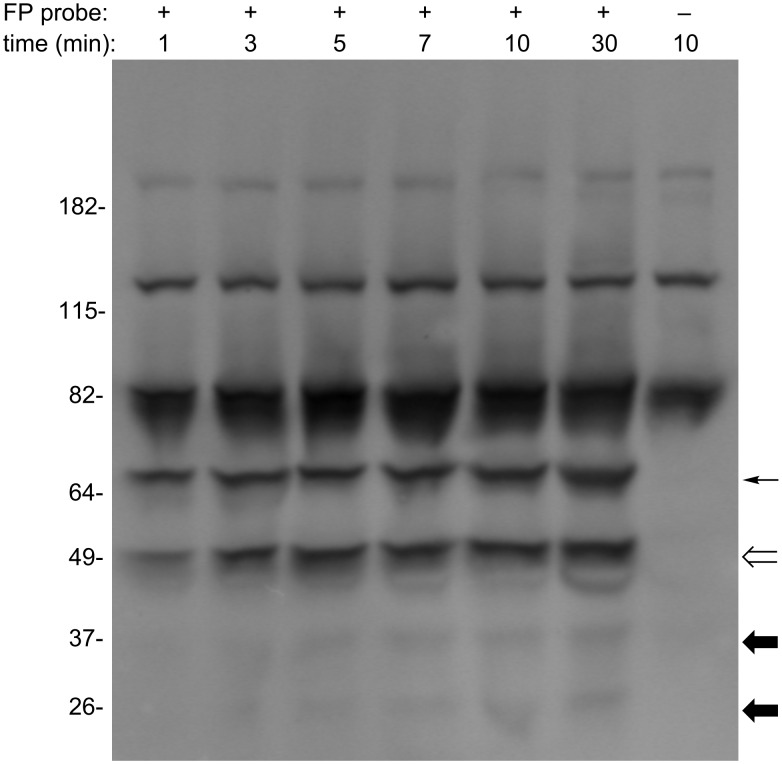
Kinetic study on FP labelling reactions. Caco-2 cell homogenates (1 mg/mL) were treated at room temperature with FP–PEG–biotin **1** (4 μM) for the indicated times, followed by the termination of reaction with 5× SDS-PAGE loading buffer. The results were analysed by 4–20% SDS-PAGE gel and streptavidin blotting. Serine hydrolases that reacted with the FP probe in a time-dependent manner are highlighted (thin, hollow and dark arrows, respectively).

An incubation-time control study is the key toward successfully monitoring the competition between FP probe **1** and a reversible substrate with a high turnover rate. Hence, over a long incubation time, the substrate will be competed out with the active enzyme covalently linked to the probe, even though the concentration of added substrate is high compared to the probe. Enzymes have different kinetic properties in the same proteome sample, and a single enzyme can have different preferences for FP probes with different linkers [6]. Thus it is important to perform kinetic studies on a specific tissue or cell line before the start of competition experiments. Toward this end, Caco-2 samples were prepared and incubated with FP–PEG–biotin **1** over different time courses ranging from 1 to 30 min (Figure 3). The sample from each trial was then separated by SDS-PAGE and blotted by streptavidin. A proteome sample was also incubated with DMSO for 10 min as a control. As all competitive exchanges between the FP probe and substrates occur within less than 15 min, data collection was limited to 30 min.

The band intensities from endogenous streptavidin-binding proteins, indicated in the control lane, did not change with incubation time. Neither did the intensity of the band around 64 kD (thin arrow), suggesting that this protein bound to the FP probe very quickly (≈50% within 1 min). In contrast, the 49 kD enzyme band (hollow arrow) showed a clear time-dependent reaction. Due to their lower expression levels, reaction of **1** with other enzymes (dark arrows) could not be detected below three minutes incubation time. Therefore, depending on the type and expression levels of serine hydrolase being investigated, different incubation times are required in order to observe a clear signal.

### Competition study

The identification of novel serine hydrolases and the screening of their covalent inhibitors against FP-biotin type probes have been extensively studied [9–11]. The general approach for serine hydrolase small molecule inhibitor development is based on the competition between an FP probe and inhibitor. To demonstrate this, a candidate inhibitor (or DMSO control) is incubated against a prepared proteome sample over a period of time. Then the sample is treated with an FP probe followed by additional incubation time. One then monitors the differential in fluorescence signal between the inhibitor and control samples. From this, selectivity and potency can be determined.

**Figure 4:**
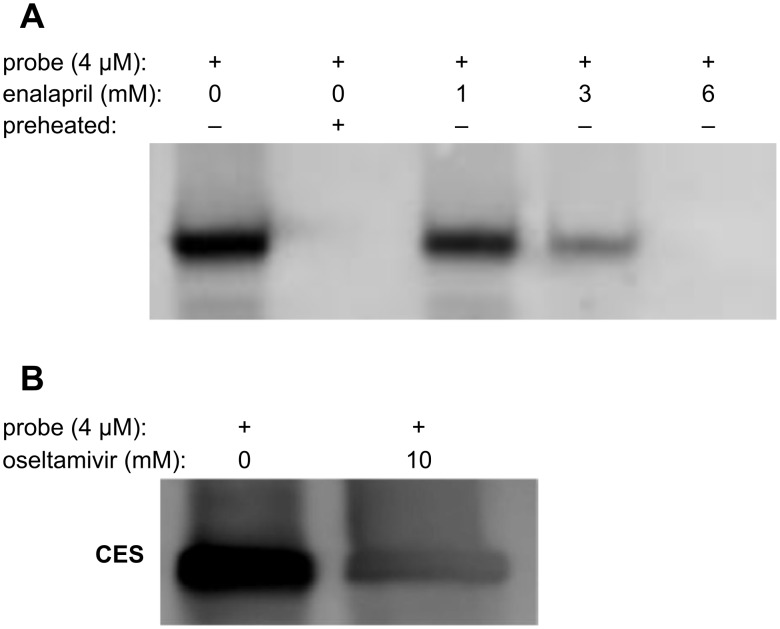
Competition assay between FP–PEG–biotin and enzyme hydrolysis reactions. (A) Enalapril at the indicated concentration was incubated in pCES (100 ng/mL) for 15 min, followed by the addition of FP probe **1** and further incubation for 15 min. Reactions were terminated by the addition of 5× SDS-PAGE loading buffer and then heated at 85 °C for 5 min. Samples were separated by SDS-PAGE and blotted by streptavidin. (B) Enzyme samples with oseltamivir were treated in the same way as enalapril.

To assess the possibility of utilizing FP probe **1** in the study of hydrolases that have substrates with high turnover rates, we chose to compete **1** against the enzyme that hydrolyzes the ester group of enalapril and oseltamivir. Enalapril and oseltamivir are commercially available prodrugs, which are converted to enalaprilat and oseltamivir carboxylate, respectively, after being absorbed in the intestine. Carboxylesterase [22], a common esterase existing in multiple human tissues, is shown to be active in the hydrolysis of both enalapril and oseltamivir [23–24]. Each is hydrolyzed at a high turnover rate. In a control run, pCES (± preheated) was treated with 4 μM FP–PEG–biotin **1** (Figure 4A, lanes 1 and 2). Then in subsequent runs, non-preheated samples of enzyme were treated with enalapril (1 mM, 3 mM) for 15 min, followed by the addition of FP–PEG–biotin (4 μM) and further incubation for 15 min at room temperature (lanes 3–5). Western blot analysis showed a clear reduction in band intensity. At 6 mM enalapril (lane 5), the band was not detectable, indicating that the competition is concentration dependent. In a similar manner, reaction of oseltamivir (10 mM) with pCES followed by the addition of FP–PEG–biotin (4 μM) showed that the pCES band intensity was reduced (Figure 4B). Thus, the reduced band intensities suggest that it is feasible to apply FP–PEG–biotin **1**, a covalent serine hydrolases probe, to the study of enzyme substrates with high turnover.

## Discussion

Fluorophosphonate probes with different analytical handles, such as rhodamine and alkyne, are being used to identify and characterize the in vivo proteome. Biotin-based probes that bind to streptavidin play an essential role in isolating and analyzing serine hydrolases. In our studies with FP–PEG–biotin probe **1**, we required large enough quantities that we decided to re-engineer the original synthesis of Kidd et al. [6]. Our major modifications involved (a) the introduction of a benzyl protecting group (in place of the TBS of the original synthesis) onto the PEG moiety to facilitate the early stages of the synthesis by providing a UV-active chromophore for easy detection; (b) introduction of a high yielding two-step iodination process, which avoids chromatography; and (c) reversing the sequence of fluoridation and amidation reactions such that the reactive FP moiety can be introduced in the last step. Overall, these modifications make for a more scalable, higher yielding sequence and importantly isolate the handling of reactive and potentially toxic FP compounds to a single step, namely the last one.

FP probes have been used mostly in the development of covalent inhibitors for pharmacologically interesting serine hydrolases due to their strong binding and rapid labeling properties. We have shown that for serine hydrolases with reversible substrates, FP probes can also be useful tools if their concentration and incubation times are properly controlled. We have demonstrated this in our studies with enalapril (6 mM) and oseltamivir (10 mM), which can each compete with FP–PEG–biotin probe **1** at a low concentration (4 μM) wherein the substrates bind reversibly to carboxylesterase and the probe binds covalently.

## Conclusion

Several steps of the original synthesis of FP–PEG–biotin probe **1** have been modified, leading to a higher overall yield and easier manipulation. Starting from common precursor **2**, our synthesis requires only four chromatographic purifications over nine steps and provides a 28.5% overall yield of **1**. In contrast, the Cravatt sequence requires six chromatographic purifications over eight steps with an overall yield of 1%. The subsequent evaluation of **1** in biological studies demonstrates its ability to interact with serine hydrolases from several proteome samples. Additionally, preliminary kinetic and competition studies of probe **1** with reversible substrates have been conducted, showing that FP probes can be utilized for the investigation of reversible substrate activities as long as sufficient care is taken to identify the time course of reaction.

## Experimental

All reagents were commercially available and used without further purification. ^1^H and ^13^C NMR spectra were obtained on Bruker 300 or Bruker 500 MHz spectrometers with CDCl_3_, *d*_6_-DMSO, or *d*_4_-methanol as solvent, and chemical shifts are reported relative to the residual solvent peak in δ (ppm). Mass spectrometry analysis was performed by using a Waters LCT time-of-flight mass spectrometry instrument. Flash column chromatography was performed with silica gel (220–240 mesh). Thin-layer chromatography (TLC) was performed on silica gel GHLF plates (250 microns) purchased from Analtech. Developed TLC plates were visualized with a UV lamp at 254 nm or by iodine staining. Extraction solutions were dried over MgSO_4_ prior to concentration.

**1-Phenyl-2,5,8,11-tetraoxatridecan-13-yl 4-methylbenzenesulfonate (4a):** This compound was made by a slight modification of the literature procedure [15]. An ice-cold solution of tetraethylene glycol monobenzyl ether (**3**; 13.9 g, 48.9 mmol; made by the procedure of Jiang et al. [14]) in 70 mL of THF was treated with KOH (9.85 g, 171.1 mmol) dissolved in 50 mL of water. A solution of *p*-toluenesulfonyl chloride (11.18 g, 58.7 mmol) in 36 mL of THF was added dropwise to the reaction mixture, which was then gradually warmed to room temperature and stirred overnight. The reaction mixture was poured into sat. aq ammonium chloride and extracted with dichloromethane (3×). The combined extracts were dried and concentrated to leave 21.11 g (98%) of **4a** as a light yellow oil: *R*_f_ 0.26 (hexanes/ethyl acetate, 1:1); ^1^H NMR (300 MHz, CDCl_3_) δ 7.80–7.77 (d, *J* = 8 Hz, 2H), 7.34–7.26 (m, 7H), 4.56 (s, 2H), 4.14 (t, *J* = 4.5 Hz, 2H), 3.69–3.58 (m, 14 H), 2.44 (s, 3H); ^13^C NMR (75 MHz, CDCl_3_) δ 144.8, 138.2, 132.9, 129.8, 128.4, 128.0, 127.7, 127.6, 73.2, 70.7, 70.6, 70.5, 69.4, 69.2, 68.6, 21.7; MS *m*/*z*: 461.0 [M + Na]^+^.

**13-Iodo-1-phenyl-2,5,8,11-tetraoxatridecane (4b):** The synthesis of **4b** utilized a procedure reported for a related compound [16]. A mixture of KI (23.97 g, 144.4 mmol) and sulfonate ester **4a** (21.11 g, 48.14 mmol) in 250 mL of acetone was heated under reflux for 18 h. After cooling to room temperature, the reaction mixture was filtered and the collected salts were rinsed with acetone. The filtrate was concentrated to an oil, which was diluted with dichloromethane. The solution was washed sequentially with sat. aq Na_2_S_2_O_3_ and brine, dried, and concentrated to give 17.44 g (92%) of **4b** as a clear oil: *R*_f_ 0.37 (hexanes/ethyl acetate, 2:1) ; ^1^H NMR (CDCl_3_, 300 MHz) δ 7.35–7.26 (m, 5H), 4.57 (s, 2H), 3.77–3.63 (m, 14H), 3.24 (t, *J* = 6.5 Hz, 2H); ^13^C NMR (75 MHz, CDCl_3_) δ 138.3, 128.4, 127.7, 127.6, 73.2, 72.0, 70.7, 70.68, 70.63, 70.2, 69.4, 3.0; MS *m*/*z*: 416.9 [M + Na]^+^.

**Diethyl (1-phenyl-2,5,8,11-tetraoxatridecan-13-yl)phosphonate (5):** Iodobenzyl polyether **4b** (17.44 g, 44.23 mmol) and triethyl phosphite (32 mL, 183.9 mmol) were mixed in an oven-dried round-bottom flask and heated under reflux for 1 h. Excess triethyl phosphite was removed under vacuum and the reaction mixture was directly loaded onto a silica-gel column, which was eluted with ethyl acetate and then methanol/ethyl acetate (5:95). Product fractions were combined and concentrated to give 16.5 g (92%) of **5** as a light yellow oil: *R*_f_ 0.11 (ethyl acetate); ^1^H NMR (500 MHz, CDCl_3_) δ 7.33–7.27 (m, 5H), 4.55 (s, 2H), 4.06 (q, *J* = 7 Hz, 4H), 3.6 (m, 14H), 2.16–2.05 (m, 2H), 1.30 (t, *J* = 7 Hz, 6H); ^13^C NMR (125 MHz, CDCl_3_) δ 138.2, 128.4, 127.7, 127.6, 73.2, 70.66, 70.63, 70.4, 70.2, 69.4, 65.1, 61.65, 61.60, 27.5, 26.5, 16.46, 16.42; MS *m*/*z*: 405.1 [M + Na]^+^.

**Diethyl (2-(2-(2-(2-hydroxyethoxy)ethoxy)ethoxy)ethyl)phosphonate (6):** A mixture of diethylphosphonate polyether **5** (3 g, 7.4 mmol), 10% Pd/C (0.3 g) and 100 mL ethanol in a 250 mL hydrogenation vessel was hydrogenated at 40 psi H_2_ for about 20 h. The reaction mixture was rapidly filtered over Celite, and concentrated under reduced pressure to give 2.23 g (96%) of **6** as a clear oil: the ^1^H and ^13^C NMR are the same as previously reported for **6** made by a different procedure [17]; MS *m*/*z*: 315.1 [M + H]^+^.

**2-(2-(2-(2-(Diethoxyphosphoryl)ethoxy)ethoxy)ethoxy)ethyl (2,5-dioxopyrrolidin-1-yl)carbonate (7):** A mixture of diethylphosphonate polyether alcohol **6** (0.5 g, 1.6 mmol), *N*,*N*-disuccinimidyl carbonate (2.04 g, 8 mmol), triethylamine (1.1 mL, 8 mmol), and anhydrous acetonitrile (4.5 mL) was stirred at room temperature for 12 h. The mixture was concentrated to an oil, which was distributed between dichloromethane and water. The organic phase was dried and concentrated to a yellow oil, which was then purified by silica-gel chromatography. Elution with dichloromethane/methanol (98:2 to 95:5) followed by pooling and concentration of product fractions provided 0.64 g (87%) of **7** as a light brown oil: the ^1^H and ^13^C NMR are the same as previously reported for **7** made by a different procedure [17]; MS *m*/*z*: 456.1 [M + H]^+^.

**2-(2-(2-(2-(Diethoxyphosphoryl)ethoxy)ethoxy)ethoxy)ethyl (5-(5-((3a*S*,4*S*,6a*R*)-2-oxohexahydro-1*H*-thieno[3,4-*d*]imidazol-4-yl)pentanamido)pentyl)carbamate (8):** A solution of in situ synthesized 5-(biotinamido)pentaneamine, trifluoroacetic acid salt, **12** (see below) in 2 mL of DMF was treated with triethylamine (0.4 mL) at 0 °C and stirred at room temperature for 10 min. Diethyl phosphonate polyether succinimidyl carbonate **7** (145 mg, 0.31 mmol) was then added and the solution was stirred at room temperature overnight. The reaction mixture was distributed between ethyl acetate and brine, and the organic phase was dried. Concentration left a brown oil, which was purified by silica-gel chromatography eluting with dichloromethane/methanol/NH_4_OH (92:8:0.5 to 90:10:1). Product fractions were pooled and concentrated to leave 324 mg (76%) of **8** as a pale white solid: ^1^H NMR (500 MHz, CDCl_3_) δ 6.66 (br s, 1H), 6.61 (br s, 1H), 6.03 (br s, 1H), 5.42 (br s, 1H), 4.46 (t, *J* = 6 Hz, 1H), 4.26 (t, *J* = 6 Hz, 1H), 4.14–4.12 (m, 2H), 4.06–4.02 (m, 4H), 3.64–3.58 (m, 12H), 3.15–3.06 (m, 7H), 2.84 (dd, *J* = 5 Hz, 13 Hz, 1H), 2.69 (d, *J* = 13 Hz, 1H), 2.13 (t, *J* = 7.3 Hz, 2H), 2.10–2.05 (m, 2H), 1.63–1.59 (m, 4H), 1.45–1.37 (m, 6H), 1.29–1.25 (m, 6H); ^13^C NMR (125 MHz, CDCl_3_) δ 173.4, 164.2, 156.6, 70.5, 70.4, 70.1, 69.6, 65.0, 63.7, 61.8, 61.7, 61.6, 60.2, 55.7, 40.6, 40.5, 39.2, 35.8, 29.5, 28.9, 28.2, 28.0, 27.4, 26.3, 25.7, 23.9, 16.4, 16.3; MS *m*/*z*: 669.2 [M + H]^+^.

**2-(2-(2-(2-(Ethoxy(hydroxy)phosphoryl)ethoxy)ethoxy)ethoxy)ethyl (5-(5-((3a*S*,4*S*,6a*R*)-2-oxohexahydro-1*H*-thieno[3,4-*d*]imidazol-4-yl)pentanamido)pentyl)carbamate (9):** A mixture of lithium azide (200 mg, 4.1 mmol), diethyl phosphonate polyether carbamate **8** (150 mg, 0.22 mmol), and 2 mL of DMF was stirred at 95 °C for 18 h. DMF was removed under vacuum to leave a yellow oil, which was diluted with water and loaded onto a column of Amberlite–IR120 (H^+^) resin. The column was eluted with DI water and the collected eluate was evacuated at 30 mm/Hg with stirring for 30 min at rt (to draw off hydrazoic acid) and then lyophilized to leave a crude yellow residue, which was purified by flash chromatography. Gradient elution with dichloromethane/methanol/NH_4_OH (70:30:2 and then 80:20:1) followed by pooling and concentration of product fractions left 0.125 g (87%) of **9** as a colorless solid: ^1^H NMR (500 MHz, CD_3_OD) δ 4.53–4.50 (m, 1H), 4.34–4.31 (m, 1H), 4.18 (t, *J* = 4.6 Hz, 2H), 3.95–3.89 (m, 2H), 3.76–3.62 (m, 12H), 3.28–3.18 (m, 3H), 3.12 (t, *J* = 7 Hz, 2H), 2.95 (dd, *J* = 4.9 Hz, 12.9 Hz, 1H), 2.73 (d, *J* = 12.9 Hz, 1H), 2.22 (t, *J* = 7.3 Hz, 2H), 1.97–1.91 (m, 2H), 1.80–1.37 (m, 12H), 1.26 (t, *J* = 7 Hz, 3H); ^13^C NMR (125 MHz, CD_3_OD) δ 174.5, 164.7, 157.3, 69.8, 69.7, 69.5, 69.2, 66.7, 63.4, 62.0, 60.2, 59.6, 55.6, 40.3, 39.7, 38.9, 35.5, 29.2, 28.7, 28.4, 28.1, 28.0, 25.6, 23.8, 15.9, 15.8; MS *m*/*z*: 641.2 [M + H]^+^.

**2-(2-(2-(2-(Ethoxy(fluoro)phosphoryl)ethoxy)ethoxy)ethoxy)ethyl (5-(5-((3a*S*,4*S*,6a*R*)-2-oxohexahydro-1*H*-thieno[3,4-*d*]imidazol-4-yl)pentanamido)pentyl)carbamate (FP–PEG–biotin; 1):** To a solution of monoethyl phosphonate polyether carbamate **9** (46 mg, 0.072 mmol) in 1 mL of anhydrous dichloromethane at −42 °C was added (diethylamino)sulfur trifluoride (DAST; 26.4 μL, 0.022 mmol). The mixture was stirred for 30 min and quenched with water at −42 °C. After stirring at room temperature for 10 min, the mixture was extracted with dichloromethane (3×). The organic phase was dried and concentrated to a yellow oil that was evacuated under high vacuum to leave 37 mg (80%) of **1**, which was used directly in biological studies: ^1^H NMR (500 MHz, CDCl_3_) δ 6.38 (br s, 1H), 5.60 (br s, 1H), 5.36 (br s, 1H), 5.29 (br s, 1H), 4.51 (m, 1H), 4.40–4.21 (m, 5H), 3.90–3.55 (m,12H), 3.30–3.11 (m, 5H), 2.92 (dd, *J* = 4.9, 12.9 Hz, 1H), 2.74 (d, *J* = 12.9 Hz, 1H), 2.35–2.18 (m, 4H), 1.85–1.40 (m, 12H); 1.31 (t, *J* = 6.0 Hz, 3H); ^13^C NMR (125 MHz, CDCl_3_) δ 173.3, 163.9, 156.6, 70.5, 70.4, 69.6, 64.2, 63.7, 63.6, 63.5, 61.8, 60.2 55.7, 40.6, 39.2, 35.8, 29.5, 28.9, 28.1, 28.0, 26.8, 25.7, 24.9, 23.8, 16.4, 16.3; ^19^F NMR (282 MHz, CDCl_3_) δ −59.4, −63.2; ^31^P NMR (121 MHz, CDCl_3_) δ 32.8, 24.0; MS *m*/*z*: 643.2 [M + H]^+^.

***tert*-Butyl (5-(5-((3a*S*,4*S*,6a*R*)-2-oxohexahydro-1*H*-thieno[3,4-*d*]imidazol-4-yl)pentanamido)pentyl)carbamate (11):** This compound was made by the procedure of Konoki et al. [18] from *tert*-butyl (5-aminopentyl)carbamate (**10**; made by the procedure of Kaur et al. [25]) and D-biotin. The ^1^H NMR spectrum is the same as previously reported [18]; ^13^C NMR (125 MHz, CDCl_3_) δ 173.3, 164.2, 156.2, 79.1, 61.8, 60.2, 55.8, 40.5, 40.3, 39.2, 36.0, 29.7, 29.1, 28.4, 28.2, 28.0, 25.8, 23.9; MS *m*/*z*: 451.1 [M + Na]^+^.

***N*-(5-Aminopentyl)-5-((3a*S*,4*S*,6a*R*)-2-oxohexahydro-1*H*-thieno[3,4-*d*]imidazol-4-yl)pentanamide (12):** This compound was made similar to the procedure of Konoki et al. [18]. To an ice-cold solution of *tert*-butyl 5-(biotinamidopentyl)carbamate (**11**; 136 mg, 0.32 mmol) in dichloromethane (2.0 mL) was added dropwise trifluoroacetic acid (1.0 mL). The cooling bath was removed and the mixture was stirred for 2 h. The solution was concentrated in vacuo to a yellow oil, which was used in the next step without further purification: ^1^H NMR (300 MHz, CD_3_OD) δ 4.45 (dd, *J* = 8.0, 5.5 Hz, 1H), 4.26 (dd, *J* = 7.5, 4.5 Hz, 1H), 3.15–3.11 (m, 3H), 2.90–2.85 (m, 1H), 2.67–2.61 (m, 3H), 2.16 (t, *J* = 7.5 Hz, 2H), 1.71–1.48 (m, 4H), 1.42–1.33 (m, 8H); MS *m*/*z*: 329.1 [M + H]^+^.

## Supporting Information

File 1 Biology experimental details and digital NMR spectra for synthesized compounds.
