# Supplementary material for: An improved synthesis of a fluorophosphonate–polyethylene glycol–biotin probe and its use against competitive substrates
Source: Beilstein J Org Chem. 2013 Jan 15;9:89–96. doi: 10.3762/bjoc.9.12 (PMC3566859; doi:10.3762/bjoc.9.12)
Supplement: File 1 — Biology experimental details and digital NMR spectra for synthesized compounds. [file Beilstein_J_Org_Chem-09-89-s001.pdf]

Supporting Information

for

An improved synthesis of a fluorophosphonate–polyethyleneglycol–biotin probe and its use against competitive substrates

Hao Xu[1,2,§], Hairat Sabit[2,§], Gordon L. Amidon[2] and H. D. Hollis Showalter[1,3,*]

Address: [1]Department of Medicinal Chemistry, University of Michigan, Ann Arbor, MI 48109-1065; [2]Department of Pharmaceutical Sciences, University of Michigan, Ann Arbor, MI 48109-1065 and [3]Vahlteich Medicinal Chemistry Core, University of Michigan, Ann Arbor, MI 48109-1065

Email: H.D. Hollis Showalter* - showalh@umich.edu

* Corresponding author

§ Equally contributing authors

Biology experimental details and digital NMR spectra for the synthesized compounds

Biological materials. Enalapril and oseltamivir were purchased from Fisher Scientific. Porcine liver esterase (EC 3.1.1.1), trypsin from bovine pancreas (EC 3.4.21.4), nucleoside phosphorylase from calf spleen (EC 2.4.2.1), and bovine serum albumin were purchased from Sigma. Cell culture reagents were obtained from Invitrogen (Rockville, MD) and cell culture supplies from Corning (Corning, NY) and Falcon (Lincoln Park, NJ).

Cells. The human colon carcinoma cell line, Caco-2, was obtained from the American Type Culture Collection (ATCC HTB37, passage numbers 18-50, Rockville, MD). The cells were maintained in Dulbecco's modified Eagle's medium containing 10% fetal bovine serum and 1% nonessential amino acids. The cells were grown in 150 × 100 mm tissue culture dishes in an atmosphere of 5% CO_2 and 90% relative humidity at 37 °C. The cells were grown for 14–16 days postconfluence before harvest.

Cell homogenate preparation. Caco-2 cell homogenate was prepared by homogenizing the Caco-2 cells with Dounce Tissue Homogenizer (Bellco Glass, Inc. Vineland, NJ) in 50 mM Tris buffer containing 0.25 M sucrose (pH 8.0). The homogenate was centrifuged for 20 min at 25,000*g*. The pellet was resuspended in 50 mM Tris buffer (pH 8.0) containing 0.5 M NaCl, and vortexed for 30 min. The resulting sample was centrifuged for 1 h at 25,000*g*. The combined supernatants were stored at −80 °C.

Tissue sample preparation. Rat tissue homogenates were prepared as follows: Immediately after surgery, rat tissues were washed three times with phosphate buffer saline (PBS). Minced samples were suspended in 50 mM Tris buffer (pH 8.0) containing

0.25 M sucrose. Samples were homogenized with Tissue-Tearor (Biospec Products, Inc., Bartlesville, OK) in an ice-water bath, and then sonicated three times on ice, 15 sec each. Each sample was centrifuged at 10,000*g* for 20 min, and the resulting pellet was resuspended in Tris buffer containing 0.5 M NaCl. After being vortexed vigorously, the suspension was sonicated briefly and centrifuged at 10,000*g* for 20 min. The combined supernatants were stored at −80 °C.

Protein labeling. Protein concentration was determined by the BCA assay and adjusted to a final concentration of 1 mg/mL in 50 mM Tris buffer (pH 8.0). The protein sample was treated with 4 μM FP-PEG-biotin probe. The reaction was incubated at room temperature for 1 min up to 1 h before being terminated by 5× sample loading buffer, and subsequently heated at 85 °C for 5 min. The treated samples were subjected to 4–20% sodium dodecyl sulfate-polyacrylamide gel electrophoresis (SDS-Page, Invitrogen Corporation). The gel was electrophoretically transferred onto Hybond PVDF membrane (Amersham, UK). The membrane was blocked in Tris buffer saline with 0.1% tween-20 (TBS-T) containing 1% casein for 1 h at room temperature, followed by incubation with streptavidin alkaline phosphatase (1:2500 dilution) (R & D, Minneapolis, MN) in TBS-T at room temperature for 1 h. The membrane was washed with TBS-T three times, 10 min for each washing. The blot was subsequently visualized with ECF substrate (GE Healthcare, NJ) on Typhoon 9200 Variable Mode Imager (GE Healthcare, NJ).

Purification of FP–PEG–biotin labeled proteins. Protein mixture was first incubated with the FP–PEG–biotin probe for 1 h at room temperature with rocking. The sample was then run through a PD-10 column (GE Healthcare, Piscataway, NJ). The eluent

was treated with SDS (0.5%) and heated for 10 min at 95 °C. After rocking with avidin agarose beads for 1 h at room temperature, the mixture was centrifuged for 3 min at 3000*g* and the supernatant was removed. The resin was washed twice each with 50 mM TBS-T buffer, and then 50 mM Tris buffer (pH 8.0). After washing, an equal volume of 2× sample buffer was added to the final remaining resin, and the mixture was heated for 10 min at 95 °C. The mixture was centrifuged at 5,000*g* for 5 min. The supernatant was then subjected to SDS-Page analysis.

Competition assay. Protein sample (1mg/mL) in 50 mM Tris buffer (pH 8.0) was incubated with enalapril (at specified concentration) for 15 min at room temperature. FP–PEG–biotin (4 μM) was then added to the sample and incubated for another 15 min before being quenched with 5× samples loading buffer, and subsequent heating at 85 °C for 5 min. Western blot analysis was conducted as described in the protein labeling section.

Digital NMR spectra

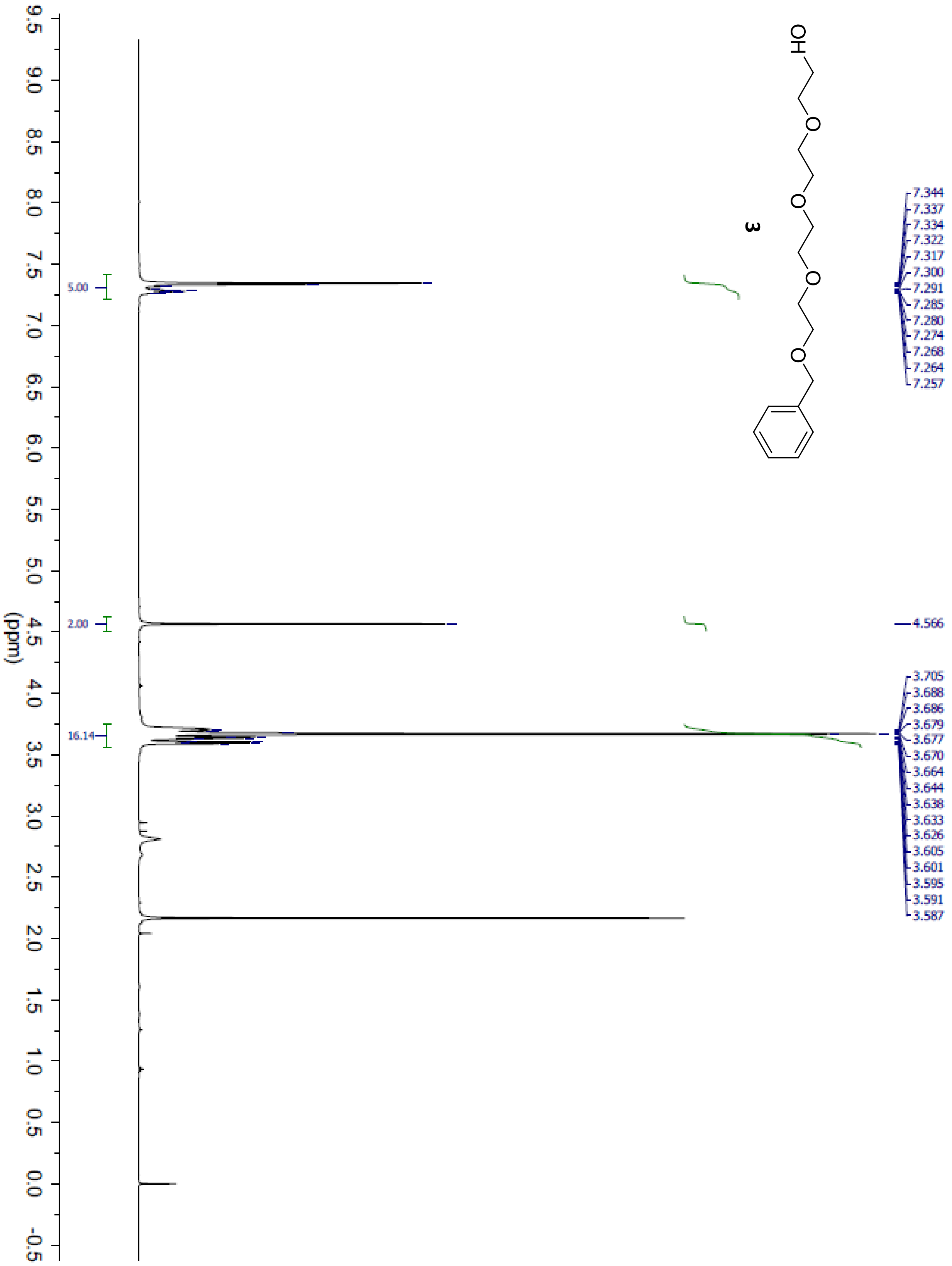

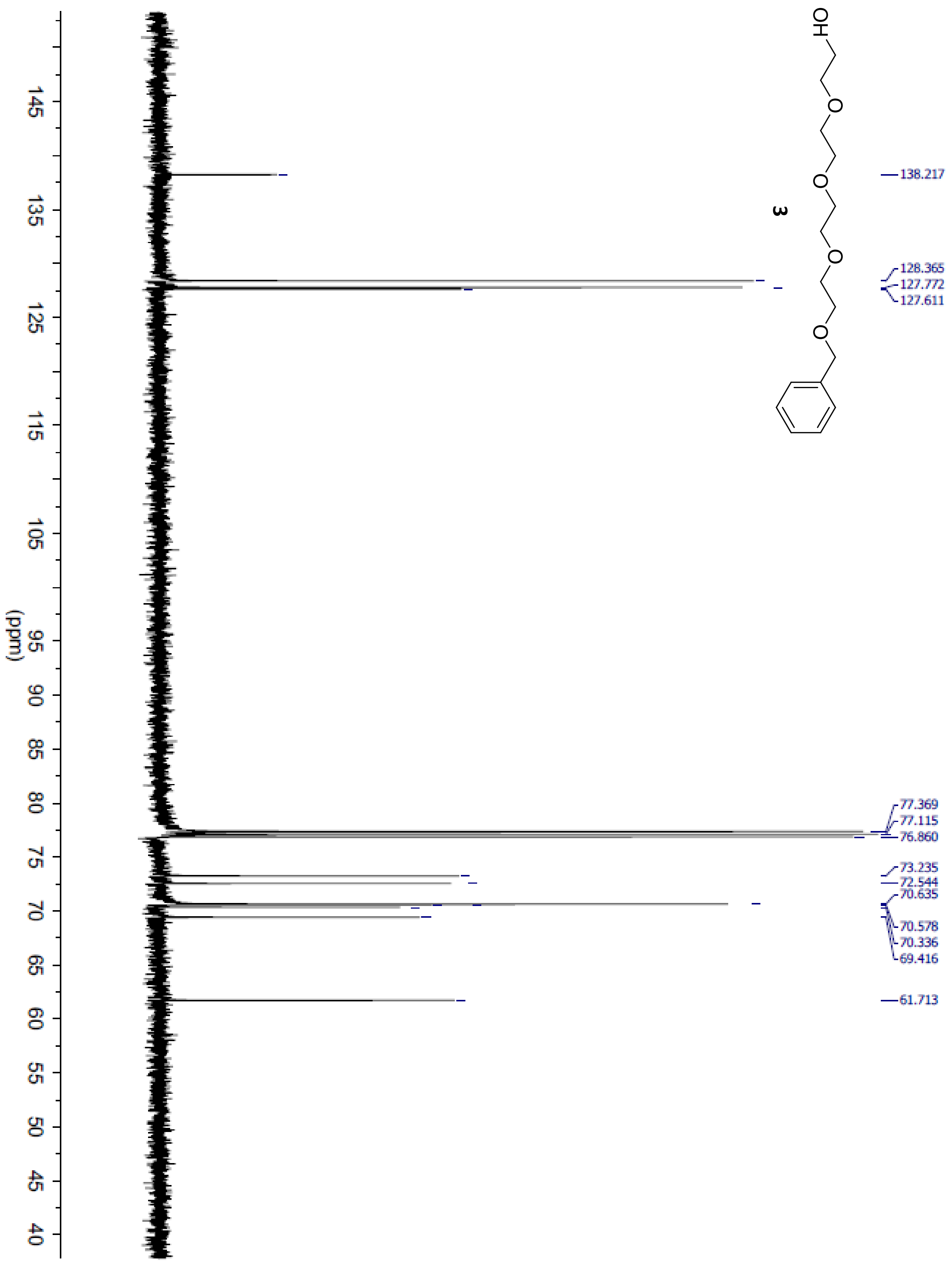

3
138.217
128.365
127.772
127.611
77.369
77.115
76.860
73.235
72.544
70.635
70.578
70.336
69.416
61.713
(ppm)

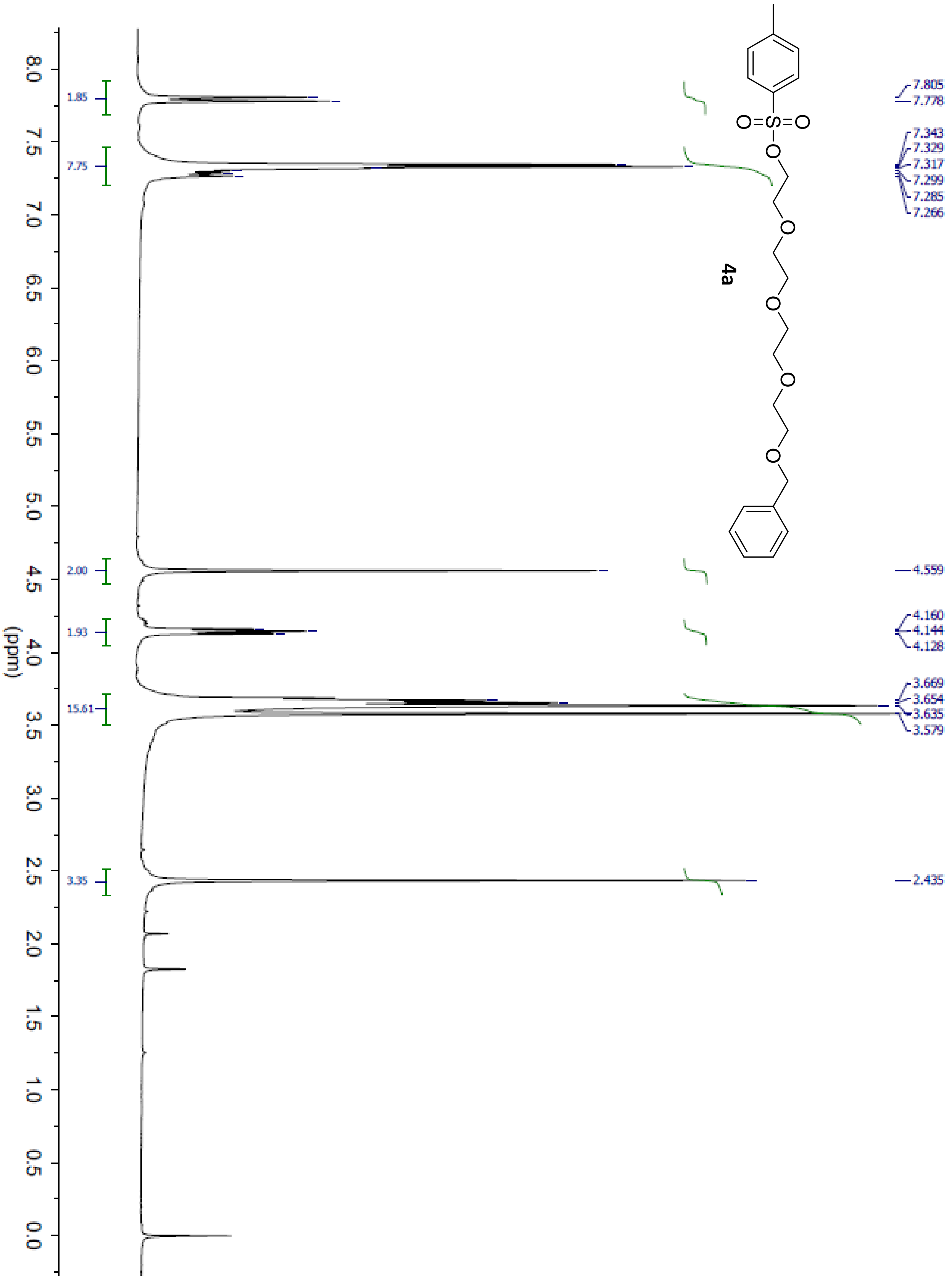
4a
(ppm)
8.0
7.5
7.0
6.5
6.0
5.5
5.0
4.5
4.0
3.5
3.0
2.5
2.0
1.5
1.0
0.5
0.0
1.85
7.75
2.00
1.93
15.61
3.35
7.805
7.778
7.343
7.329
7.317
7.299
7.285
7.266
4.559
4.160
4.144
4.128
3.669
3.654
3.635
3.579
2.435

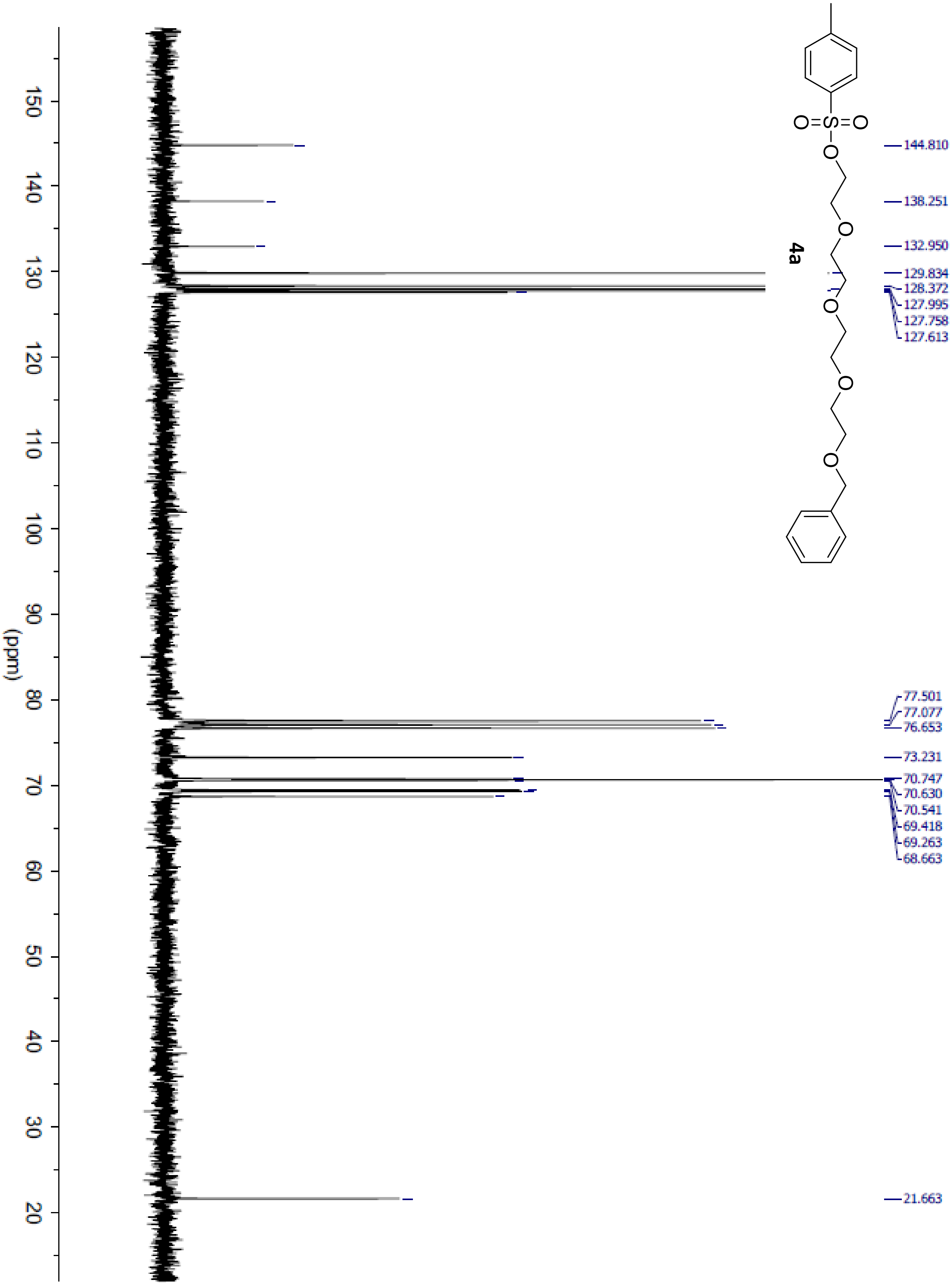

4a
144.810
138.251
132.950
129.834
128.372
127.995
127.758
127.613
77.501
77.077
76.653
73.231
70.747
70.630
70.541
69.418
69.263
68.663
21.663
(ppm)

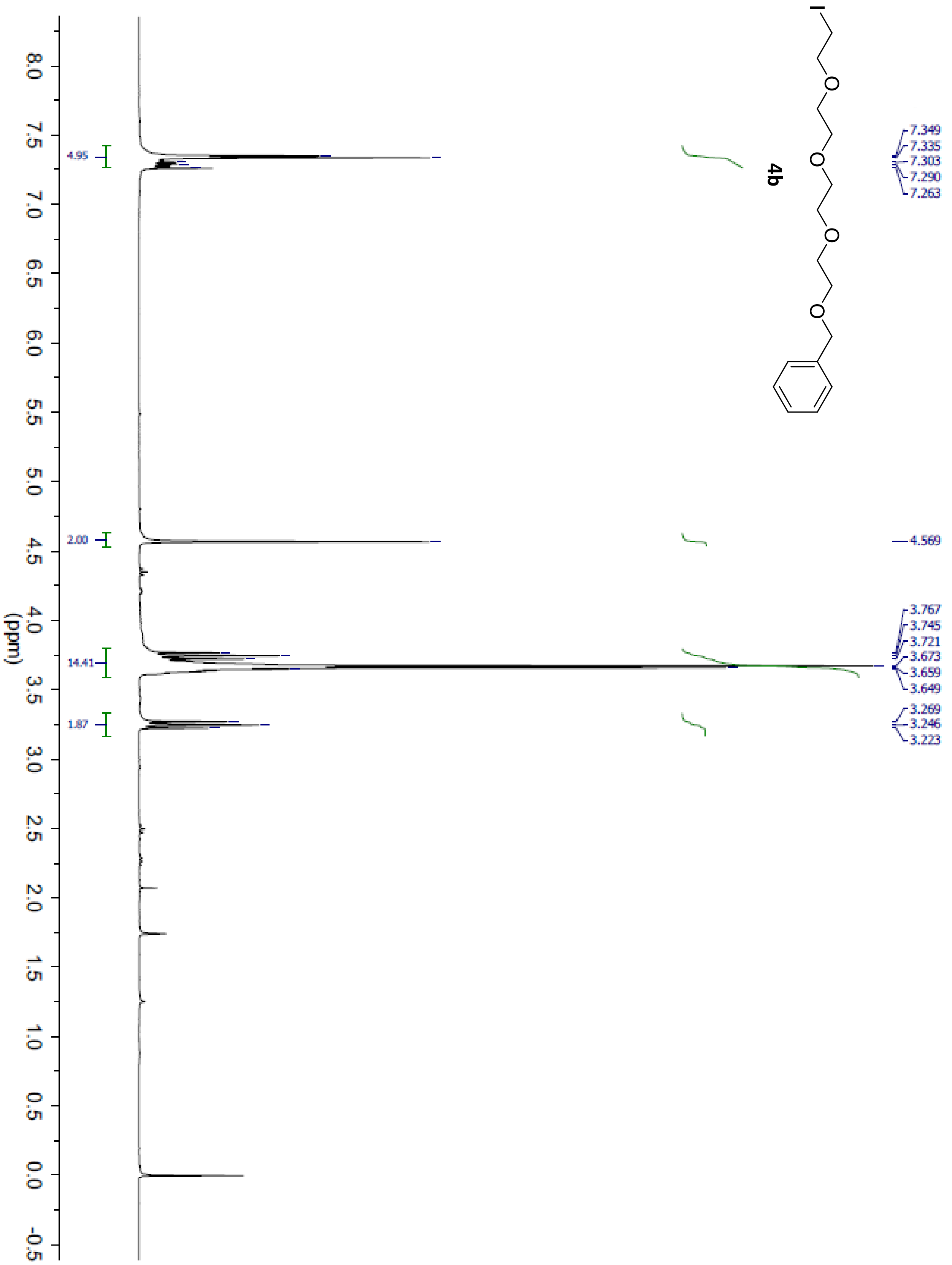
4b
7.349
7.335
7.303
7.290
7.263
4.569
3.767
3.745
3.721
3.673
3.659
3.649
3.269
3.246
3.223
4.95
2.00
14.41
1.87
(ppm)

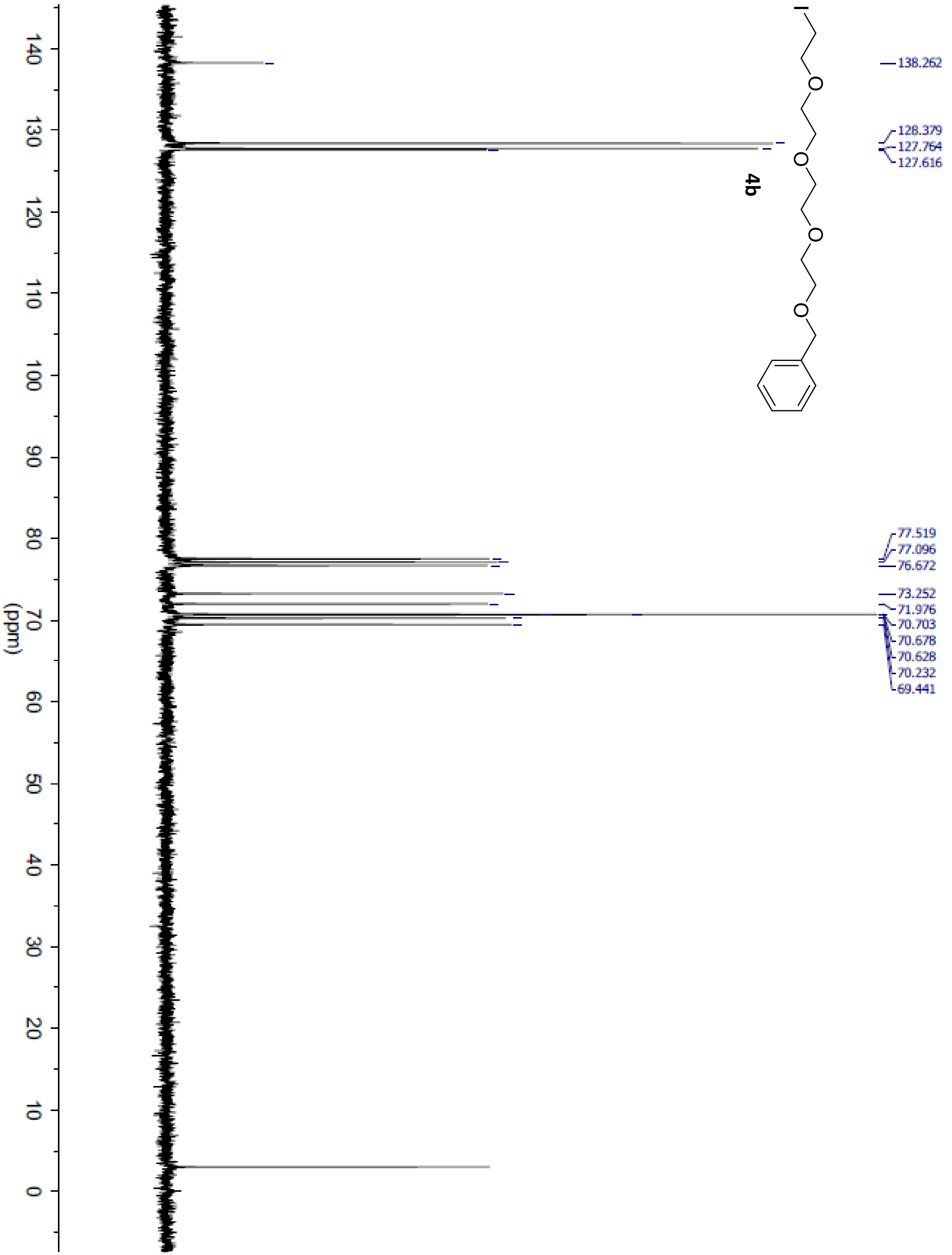

4b
138.262
128.379
127.764
127.616
77.519
77.096
76.672
73.252
71.976
70.703
70.678
70.628
70.232
69.441
(ppm)

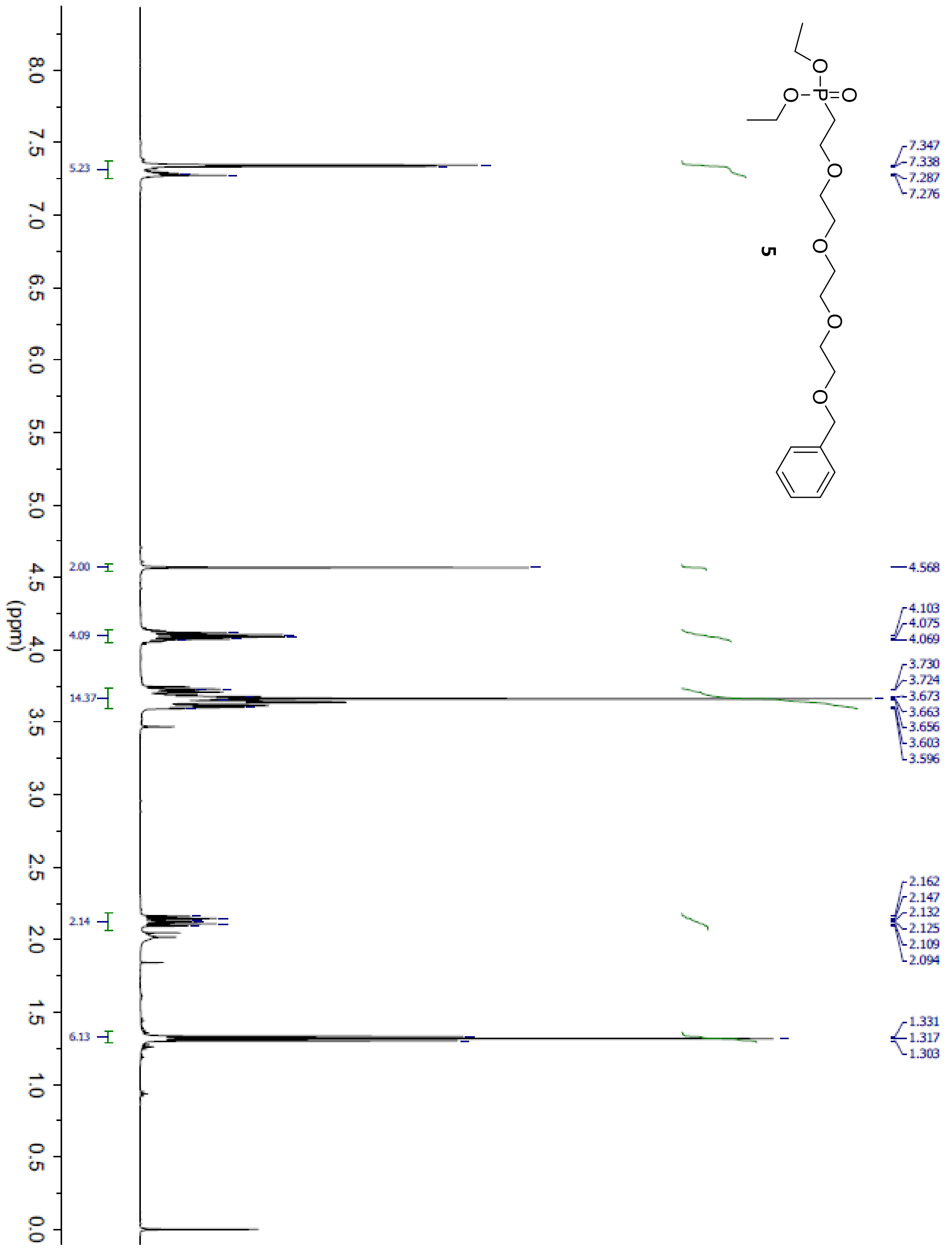
5
(ppm)
5.23
2.00
4.09
14.37
2.14
6.13
7.347
7.338
7.287
7.276
4.568
4.103
4.075
4.069
3.730
3.724
3.673
3.663
3.656
3.603
3.596
2.162
2.147
2.132
2.125
2.109
2.094
1.331
1.317
1.303

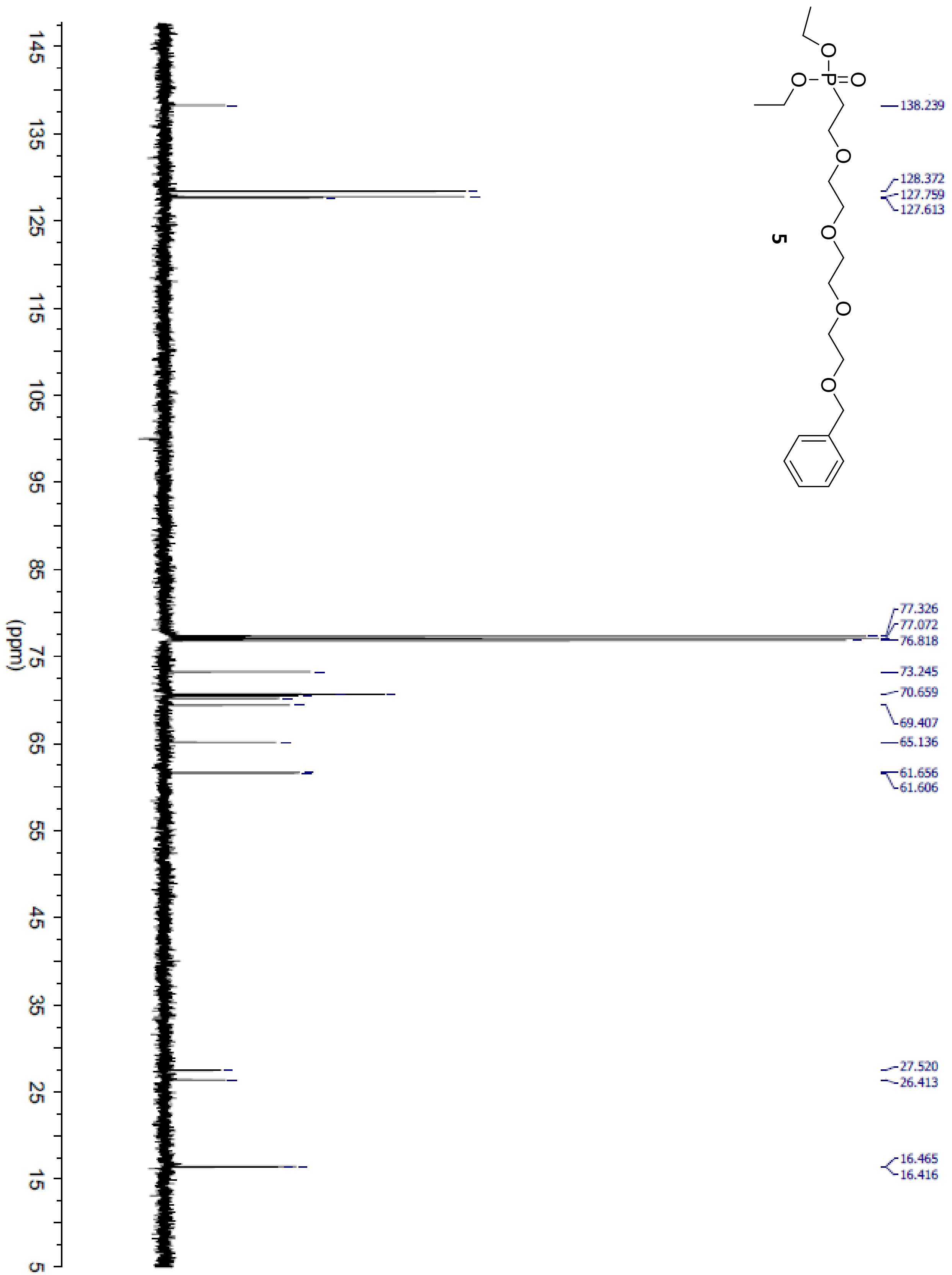

5
138.239
128.372
127.759
127.613
77.326
77.072
76.818
73.245
70.659
69.407
65.136
61.656
61.606
27.520
26.413
16.465
16.416
(ppm)
145
135
125
115
105
95
85
75
65
55
45
35
25
15
5

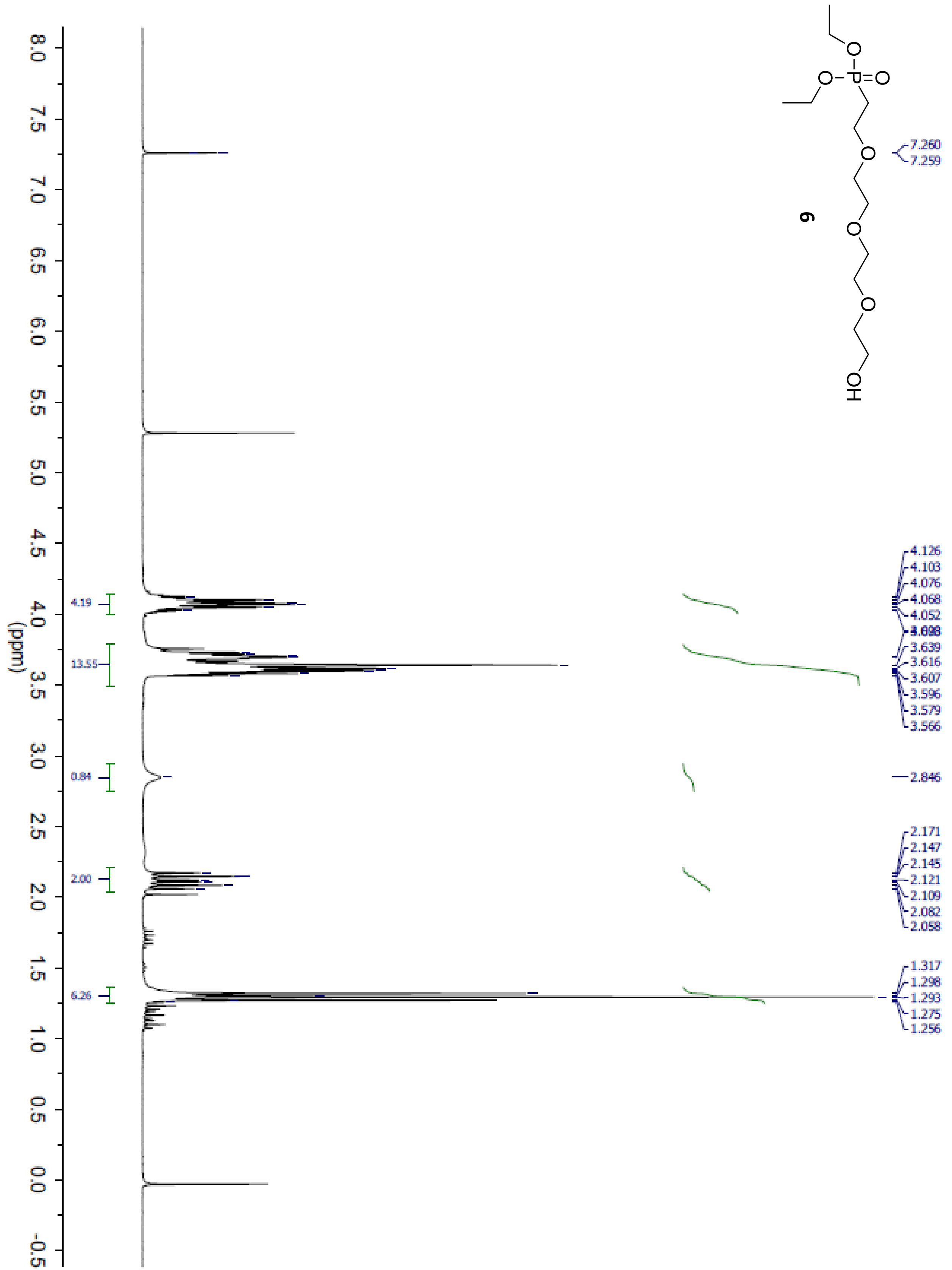

6
OH
O
P
7.260
7.259
4.126
4.103
4.076
4.068
4.052
3.639
3.616
3.607
3.596
3.579
3.566
2.846
2.171
2.147
2.145
2.121
2.109
2.082
2.058
1.317
1.298
1.293
1.275
1.256
4.19
13.55
0.84
2.00
6.26
(ppm)

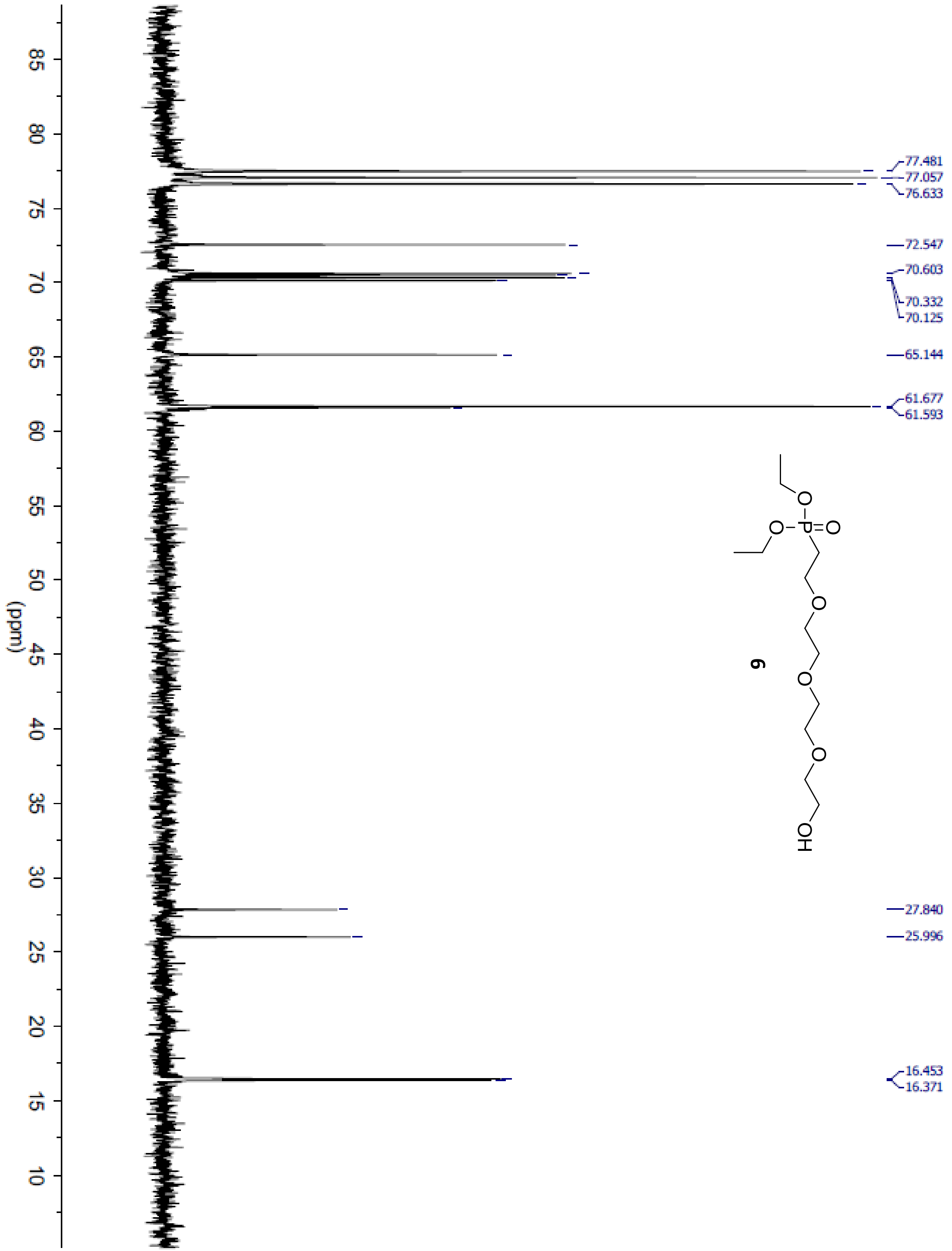
77.481
77.057
76.633
72.547
70.603
70.332
70.125
65.144
61.677
61.593
27.840
25.996
16.453
16.371
(ppm)
6
OH

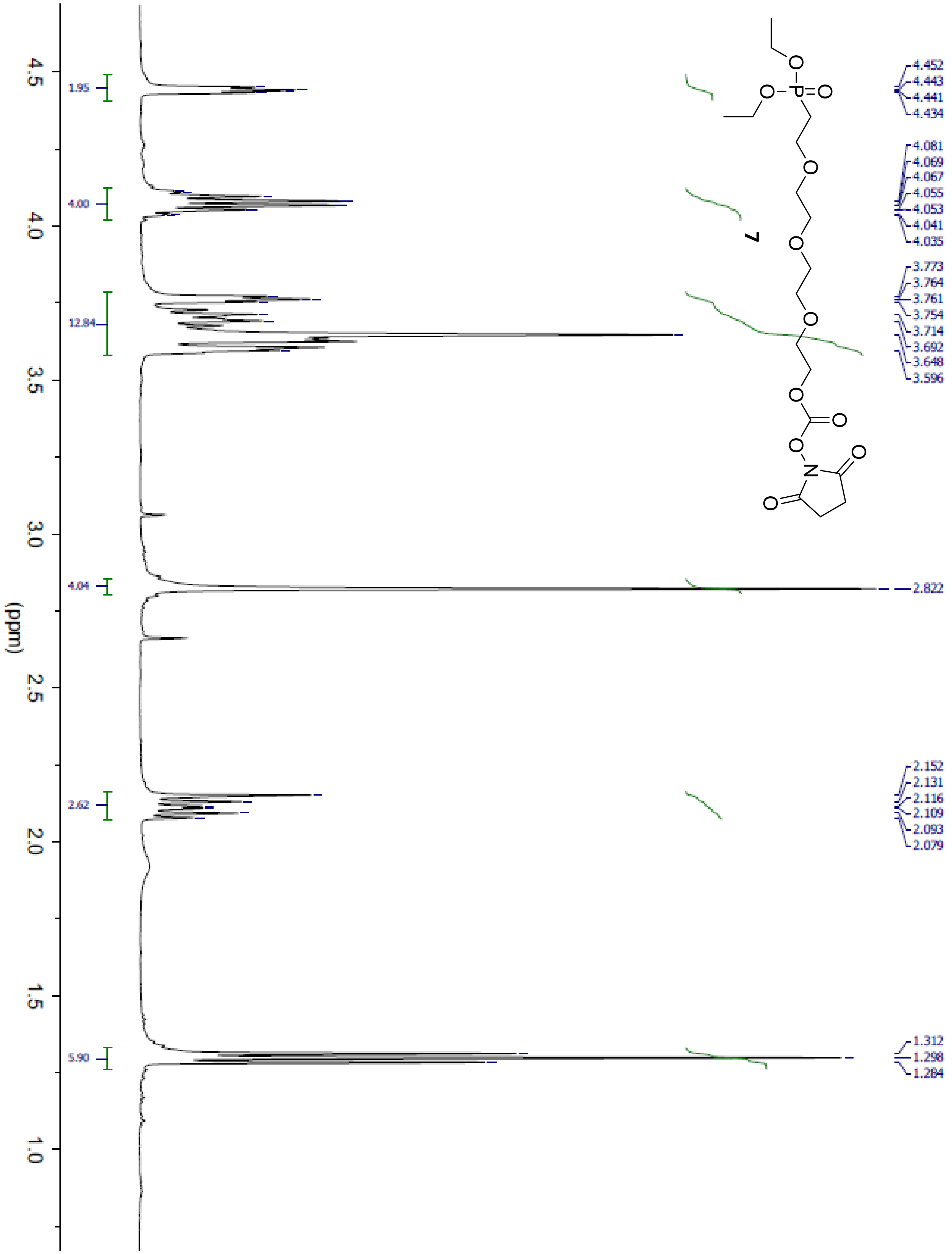
4.452
4.443
4.441
4.434
4.081
4.069
4.067
4.055
4.053
4.041
4.035
3.773
3.764
3.761
3.754
3.714
3.692
3.648
3.596
2.822
2.152
2.131
2.116
2.109
2.093
2.079
1.312
1.298
1.284
1.95
4.00
12.84
4.04
2.62
5.90
4.5
4.0
3.5
3.0
2.5
2.0
1.5
1.0
(ppm)
7

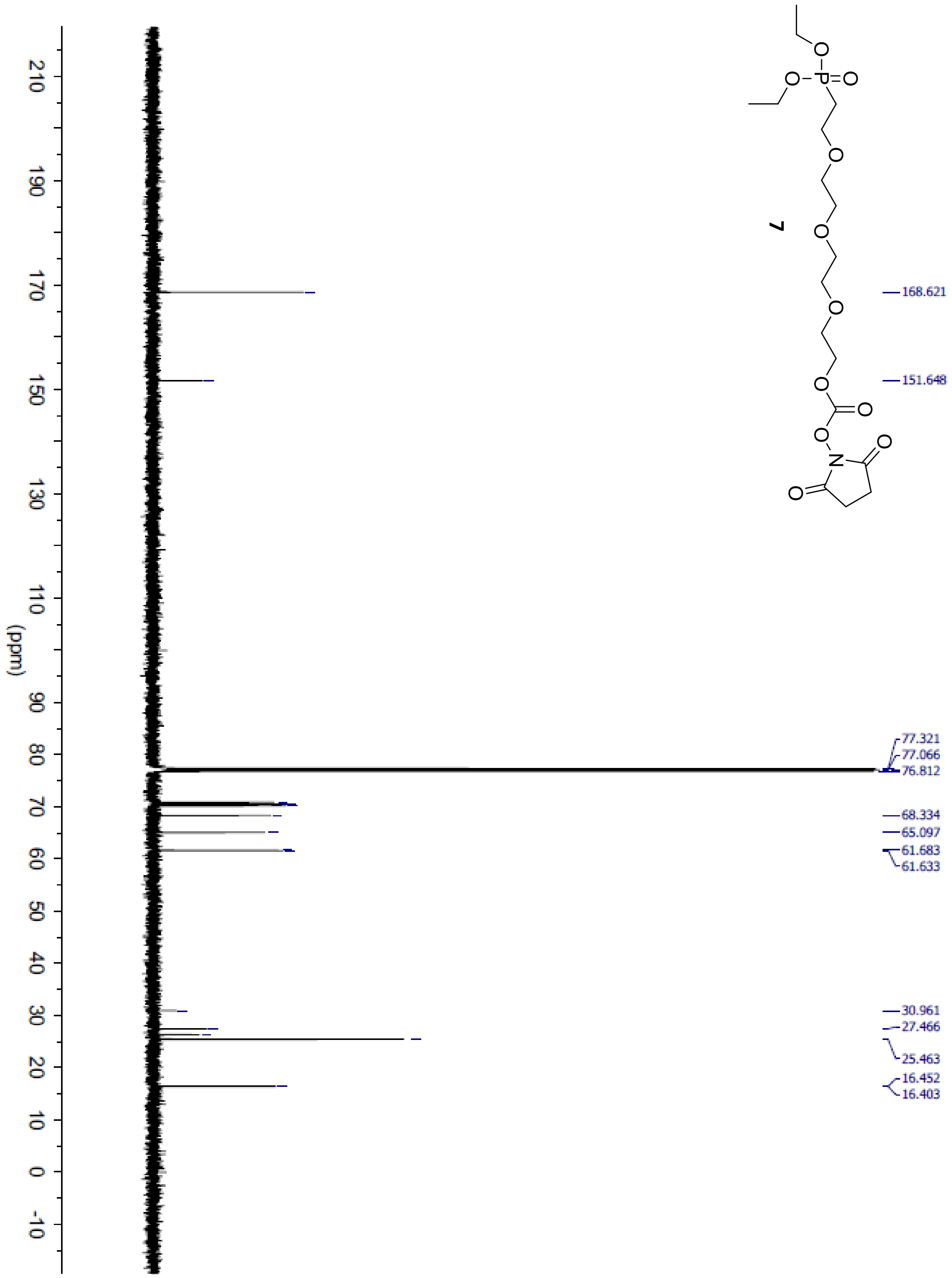
7
168.621
151.648
77.321
77.066
76.812
68.334
65.097
61.683
61.633
30.961
27.466
25.463
16.452
16.403
(ppm)
210
190
170
150
130
110
90
80
70
60
50
40
30
20
10
0
-10

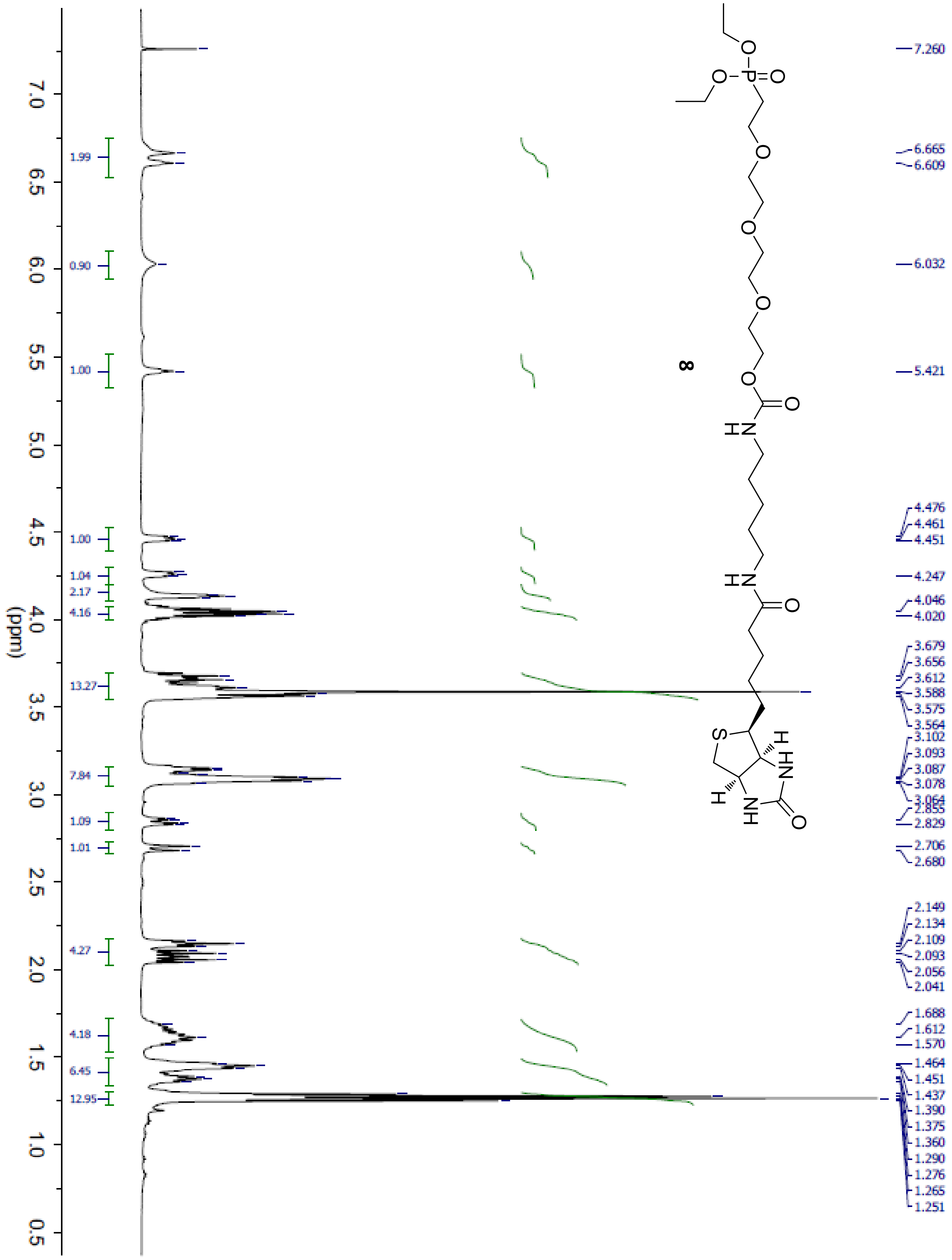
8
(ppm)

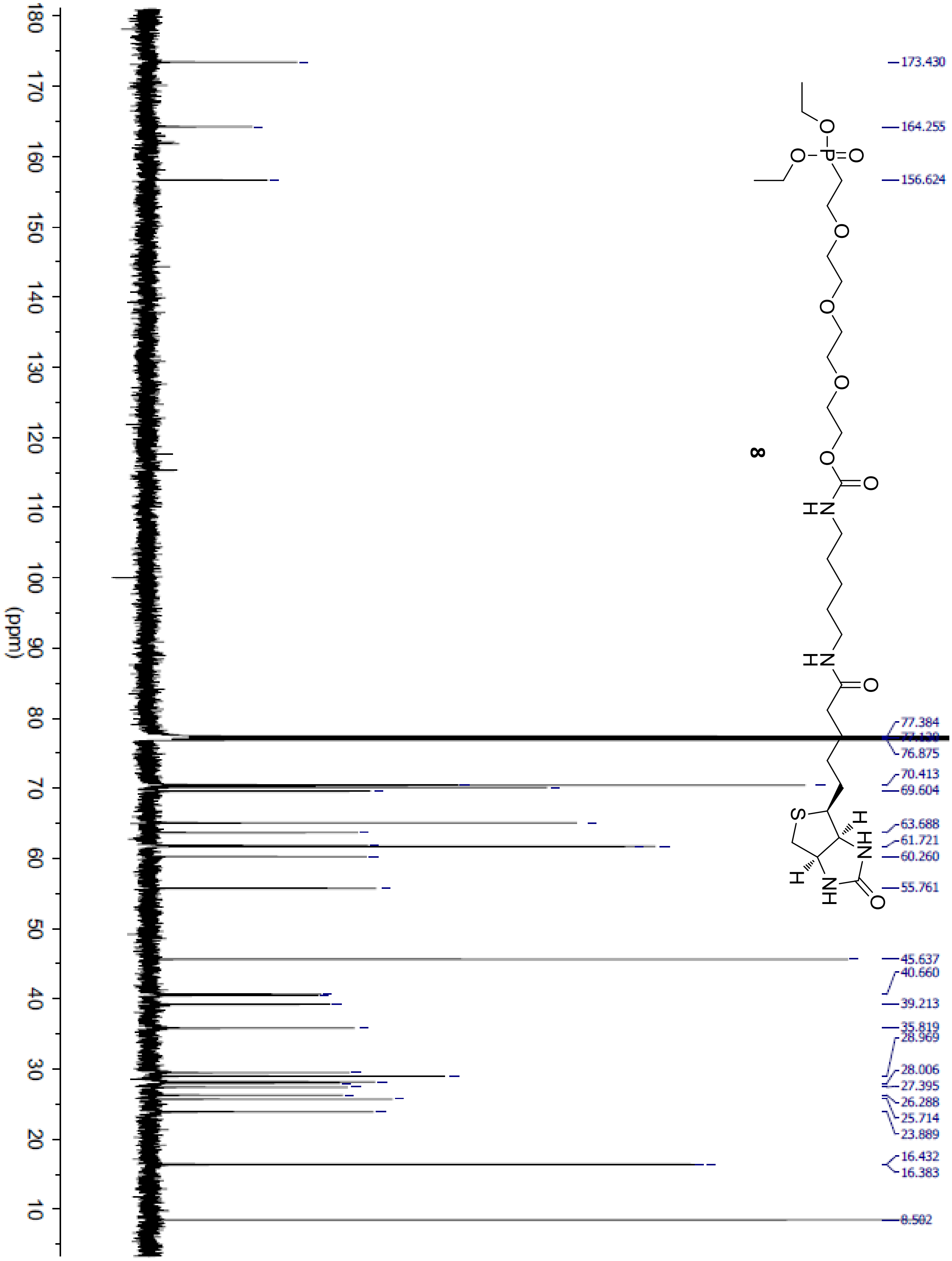

173.430
164.255
156.624
77.384
76.875
70.413
69.604
63.688
61.721
60.260
55.761
45.637
40.660
39.213
35.819
28.969
28.006
27.395
26.288
25.714
23.889
16.432
16.383
8.502
8
(ppm)

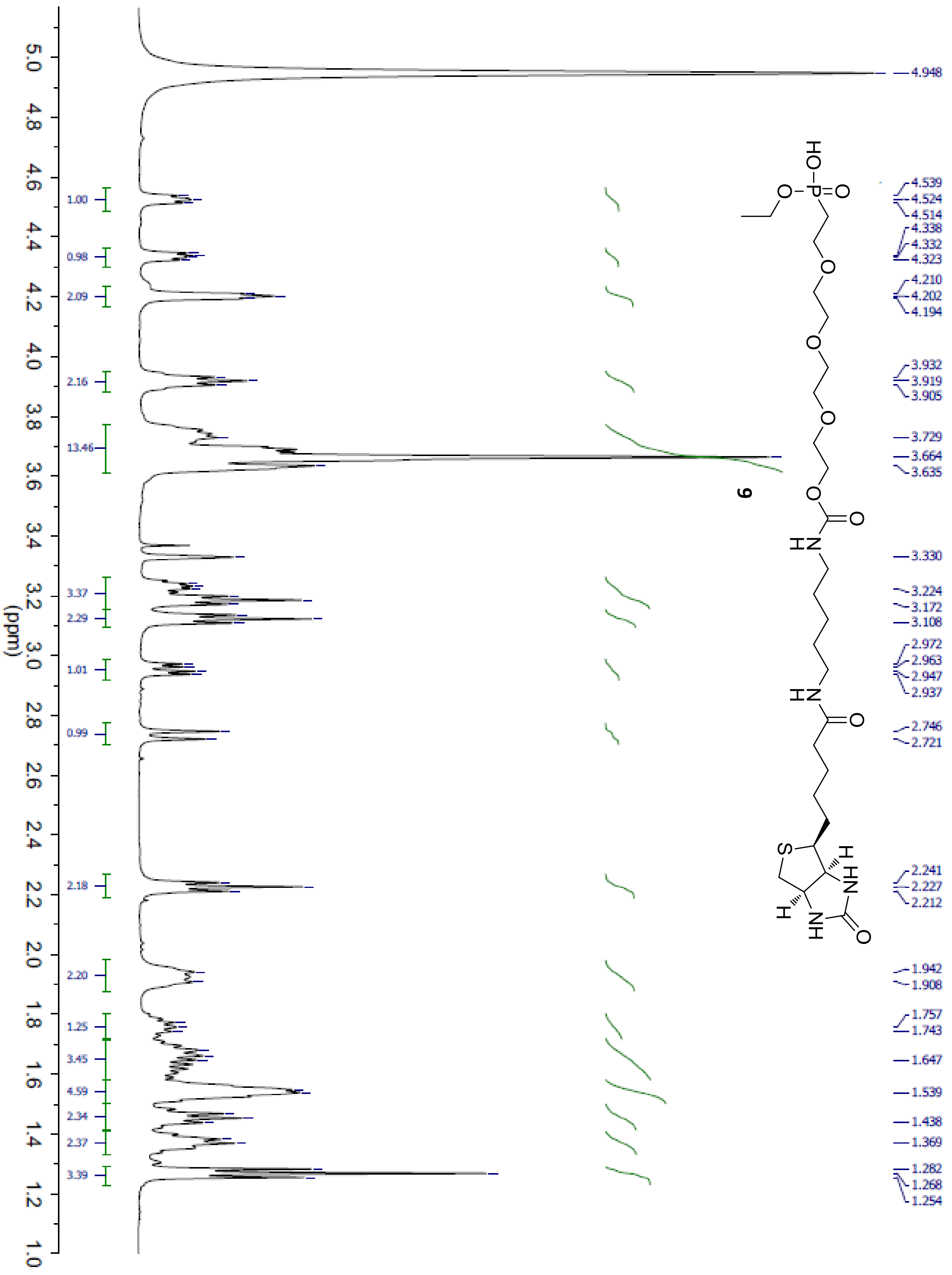
4.948
4.539
4.524
4.514
4.338
4.332
4.323
4.210
4.202
4.194
3.932
3.919
3.905
3.729
3.664
3.635
3.330
3.224
3.172
3.108
2.972
2.963
2.947
2.937
2.746
2.721
2.241
2.227
2.212
1.942
1.908
1.757
1.743
1.647
1.539
1.438
1.369
1.282
1.268
1.254
1.00
0.98
2.09
2.16
13.46
3.37
2.29
1.01
0.99
2.18
2.20
1.25
3.45
4.59
2.34
2.37
3.39
5.0
4.8
4.6
4.4
4.2
4.0
3.8
3.6
3.4
3.2
3.0
2.8
2.6
2.4
2.2
2.0
1.8
1.6
1.4
1.2
1.0
(ppm)
9

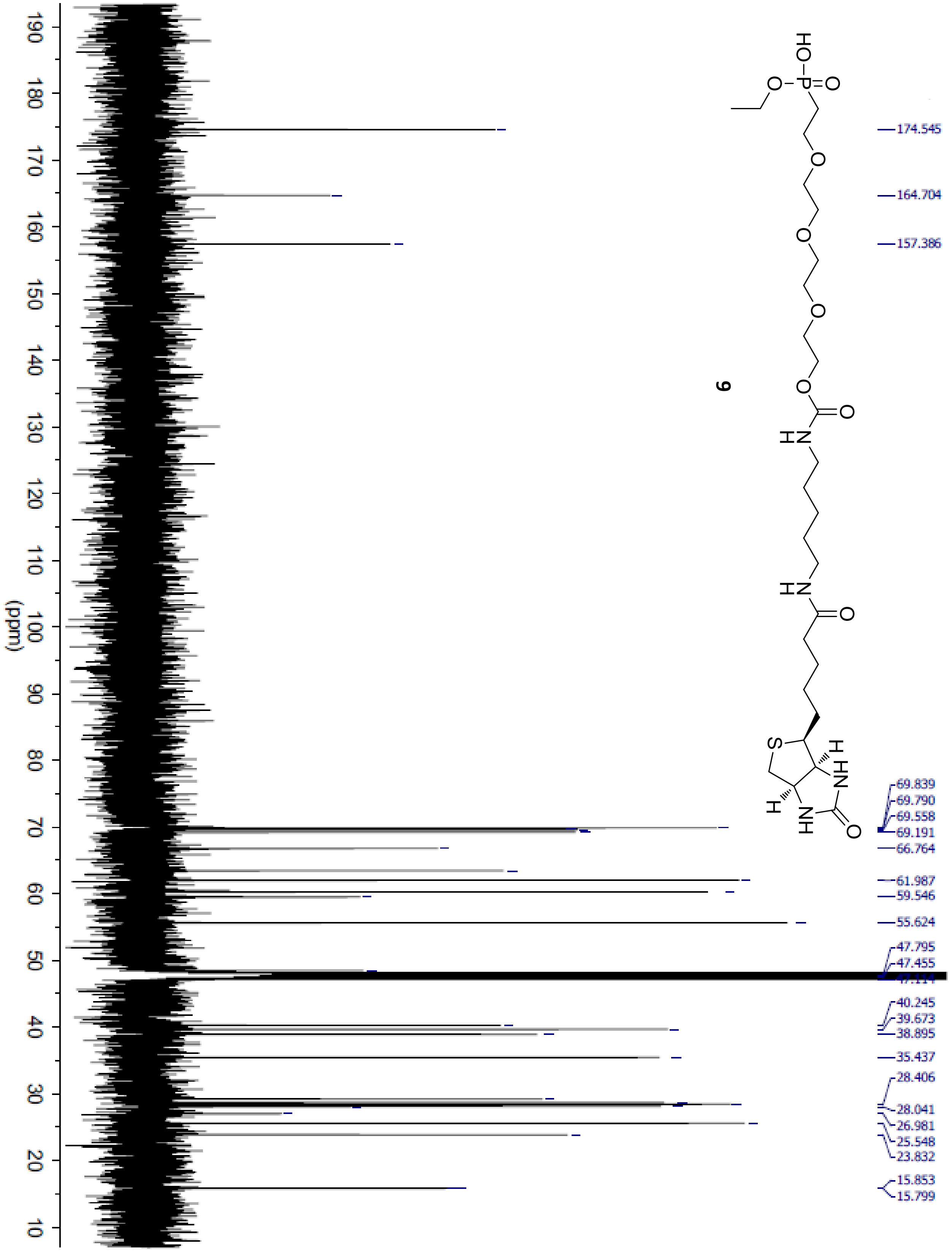
9
HO
P
O
N
H
S
HN
NH
(ppm)
190
180
170
160
150
140
130
120
110
100
90
80
70
60
50
40
30
20
10
174.545
164.704
157.386
69.839
69.790
69.558
69.191
66.764
61.987
59.546
55.624
47.795
47.455
40.245
39.673
38.895
35.437
28.406
28.041
26.981
25.548
23.832
15.853
15.799

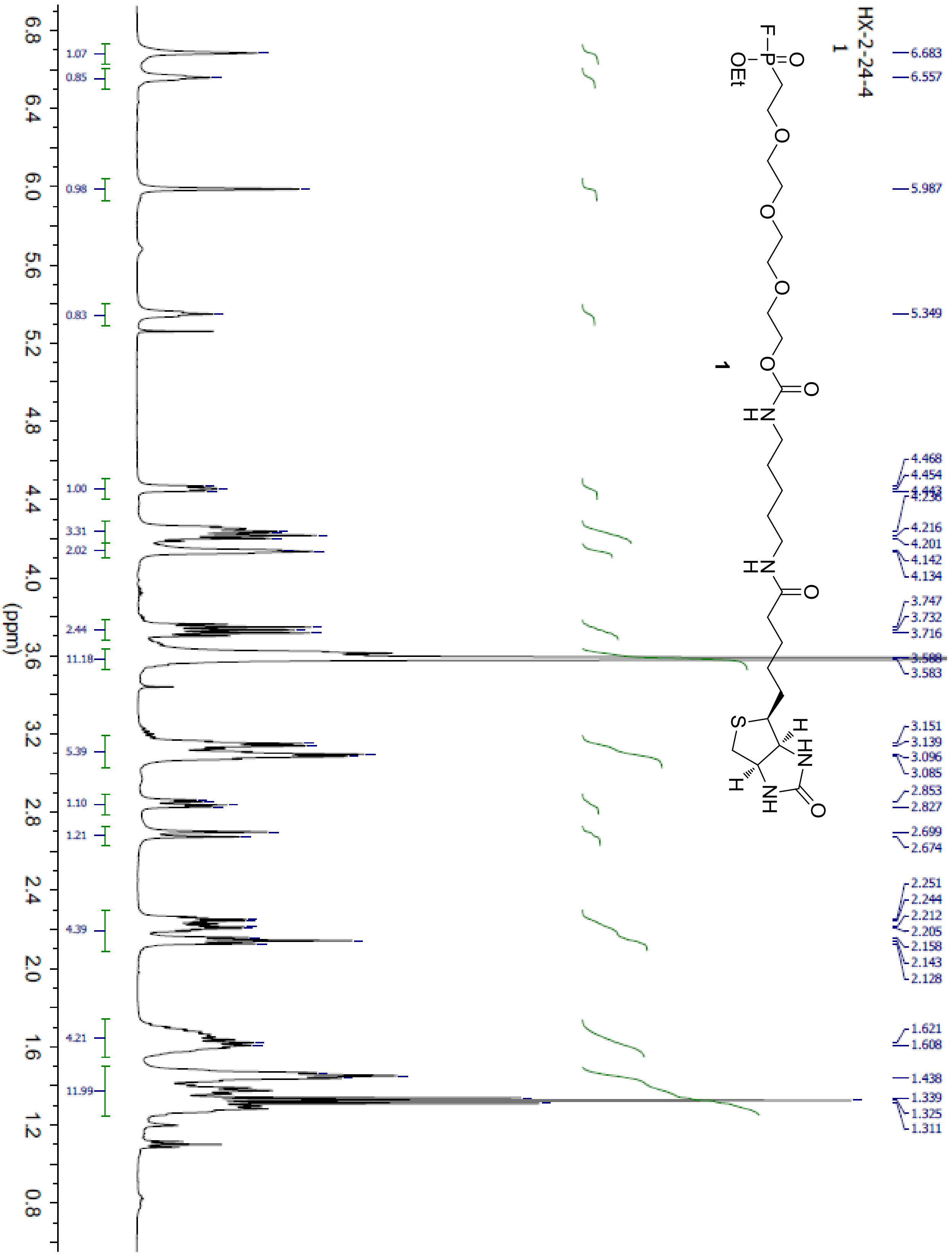

HX-2-24-4
1
6.683
6.557
5.987
5.349
4.468
4.454
4.443
4.236
4.216
4.201
4.142
4.134
3.747
3.732
3.716
3.588
3.583
3.151
3.139
3.096
3.085
2.853
2.827
2.699
2.674
2.251
2.244
2.212
2.205
2.158
2.143
2.128
1.621
1.608
1.438
1.339
1.325
1.311
1.07
0.85
0.98
0.83
1.00
3.31
2.02
2.44
11.18
5.39
1.10
1.21
4.39
4.21
11.99
(ppm)

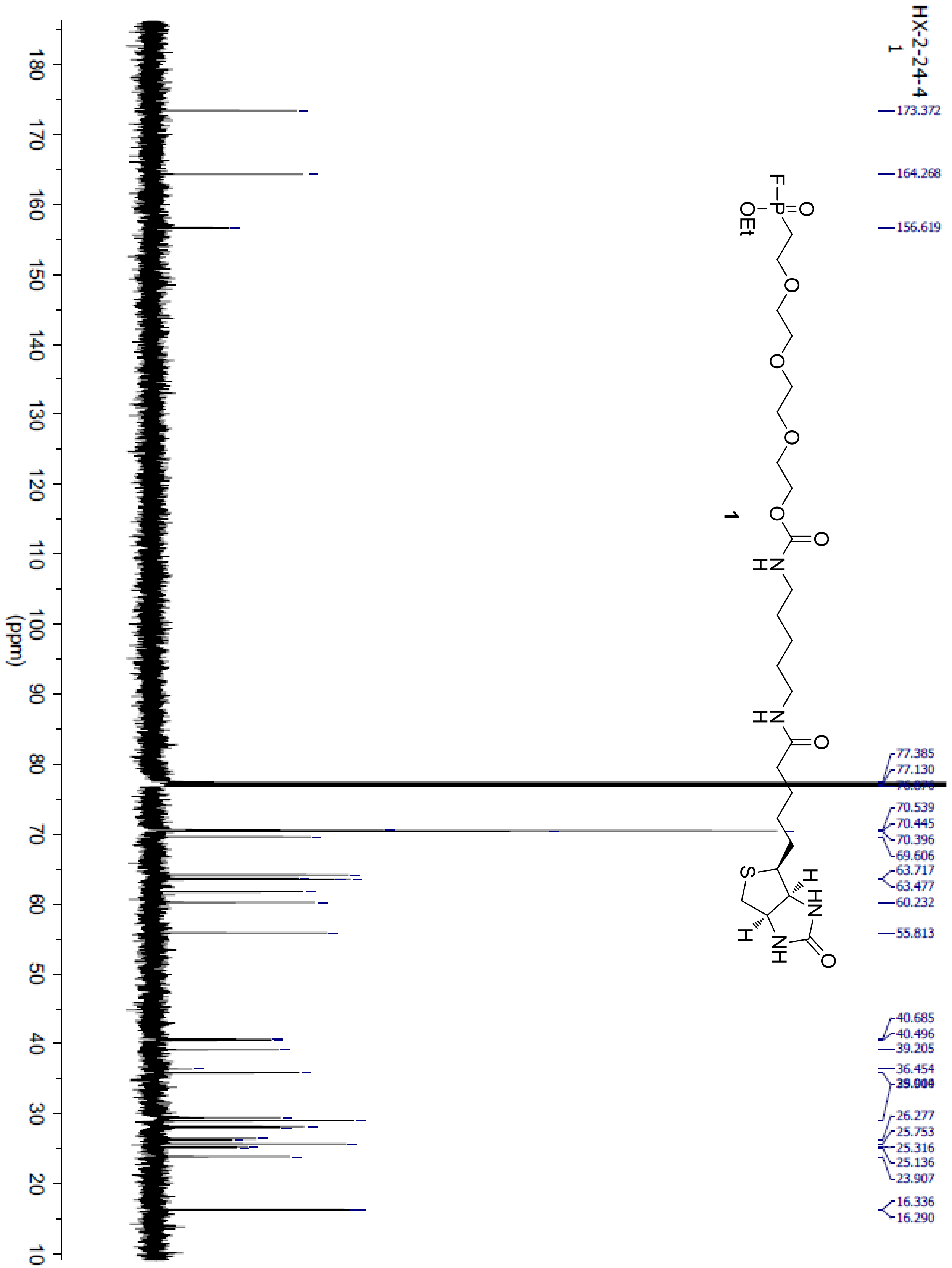

HX-2-24-4
1
173.372
164.268
156.619
77.385
77.130
70.539
70.445
70.396
69.606
63.717
63.477
60.232
55.813
40.685
40.496
39.205
36.454
26.277
25.753
25.316
25.136
23.907
16.336
16.290
(ppm)

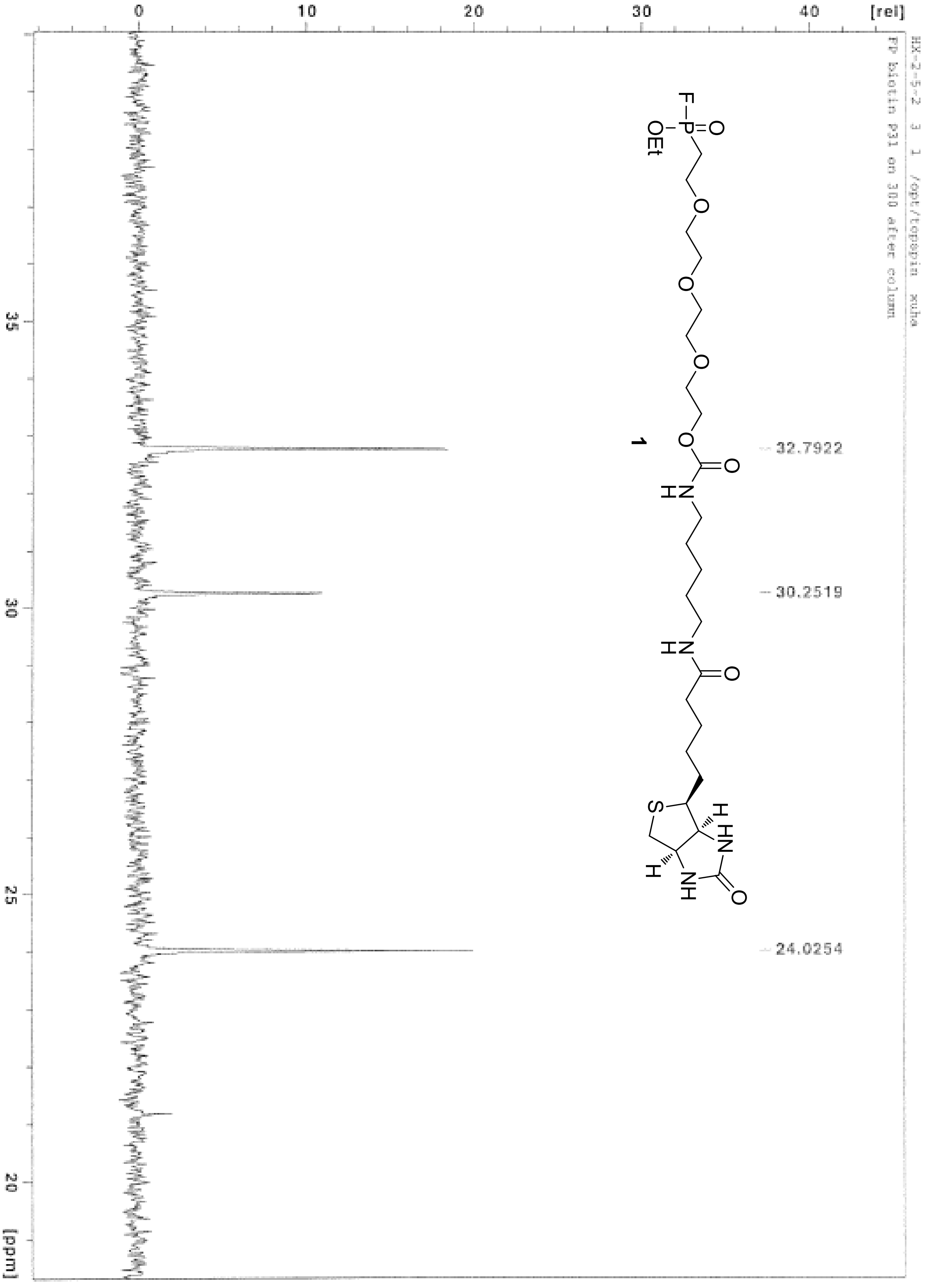
HX-2-5-2 3 1 /opt/topspin xuha
FP biotin P31 on 300 after column
1
32.7922
30.2519
24.0254
[rel]
[ppm]

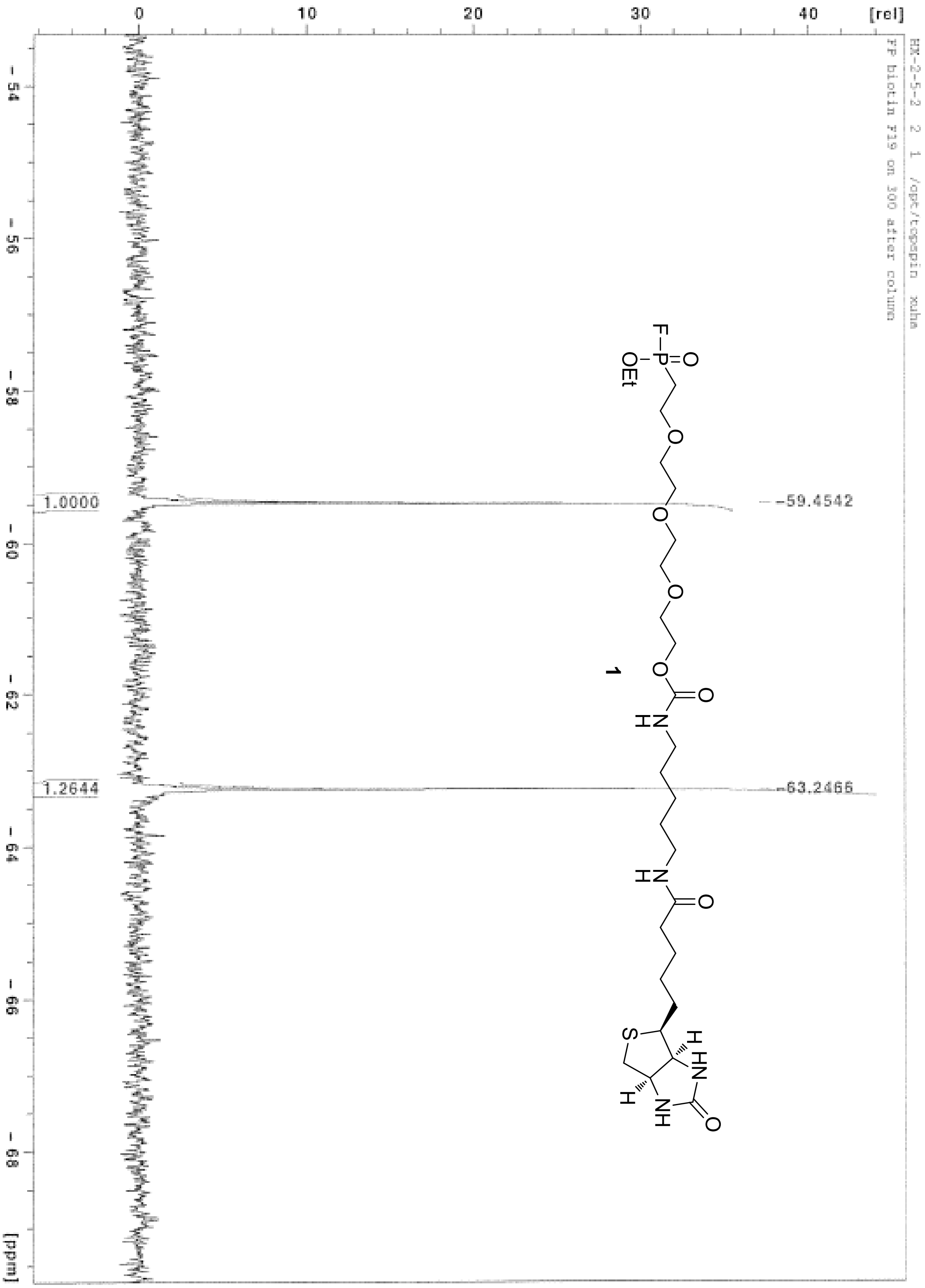
HX-2-5-2 2 1 /opt/topspin xuhe
FF biotin F19 on 300 after column
1
1.0000
-59.4542
1.2644
-63.2466
[rel]
[ppm]

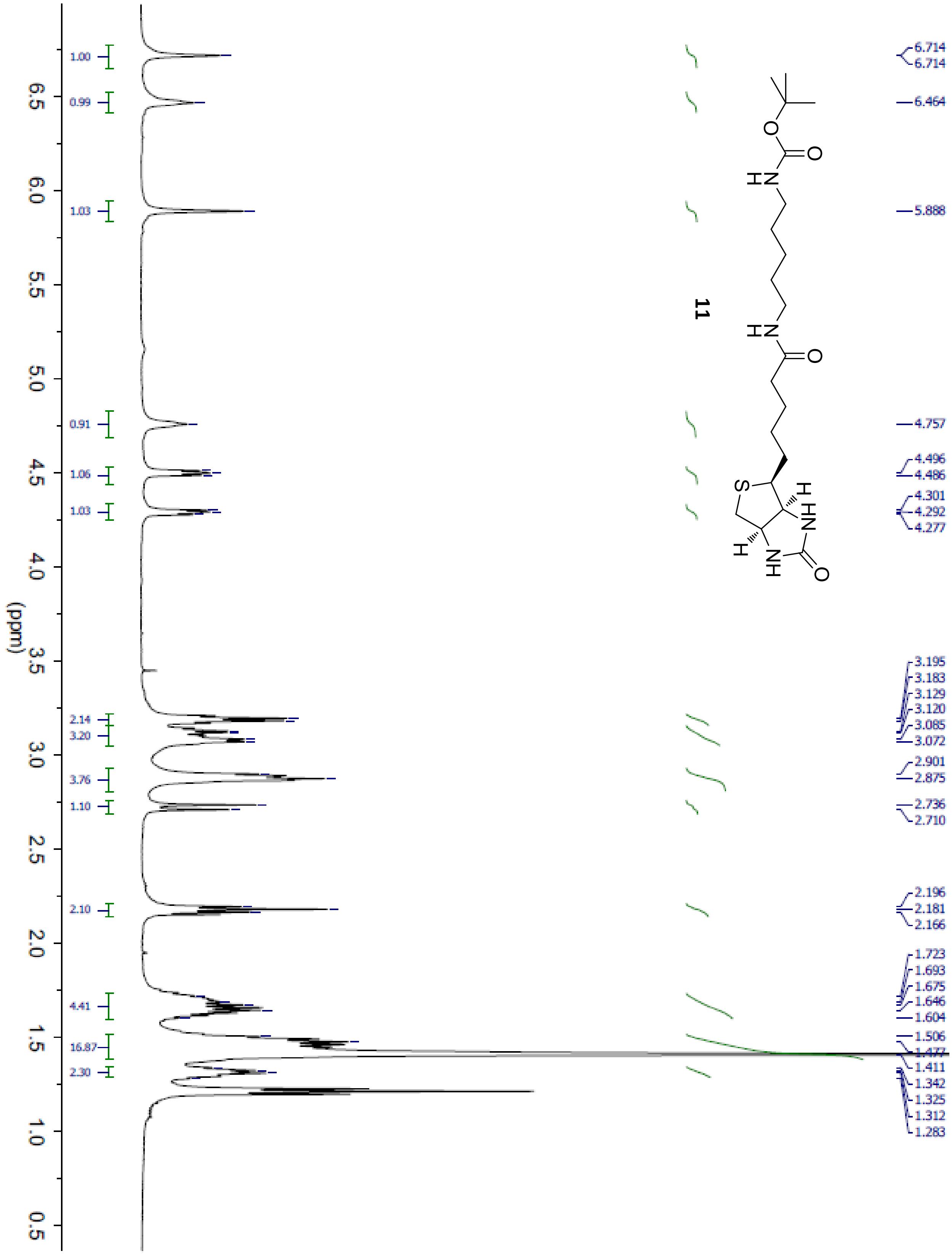
11
(ppm)

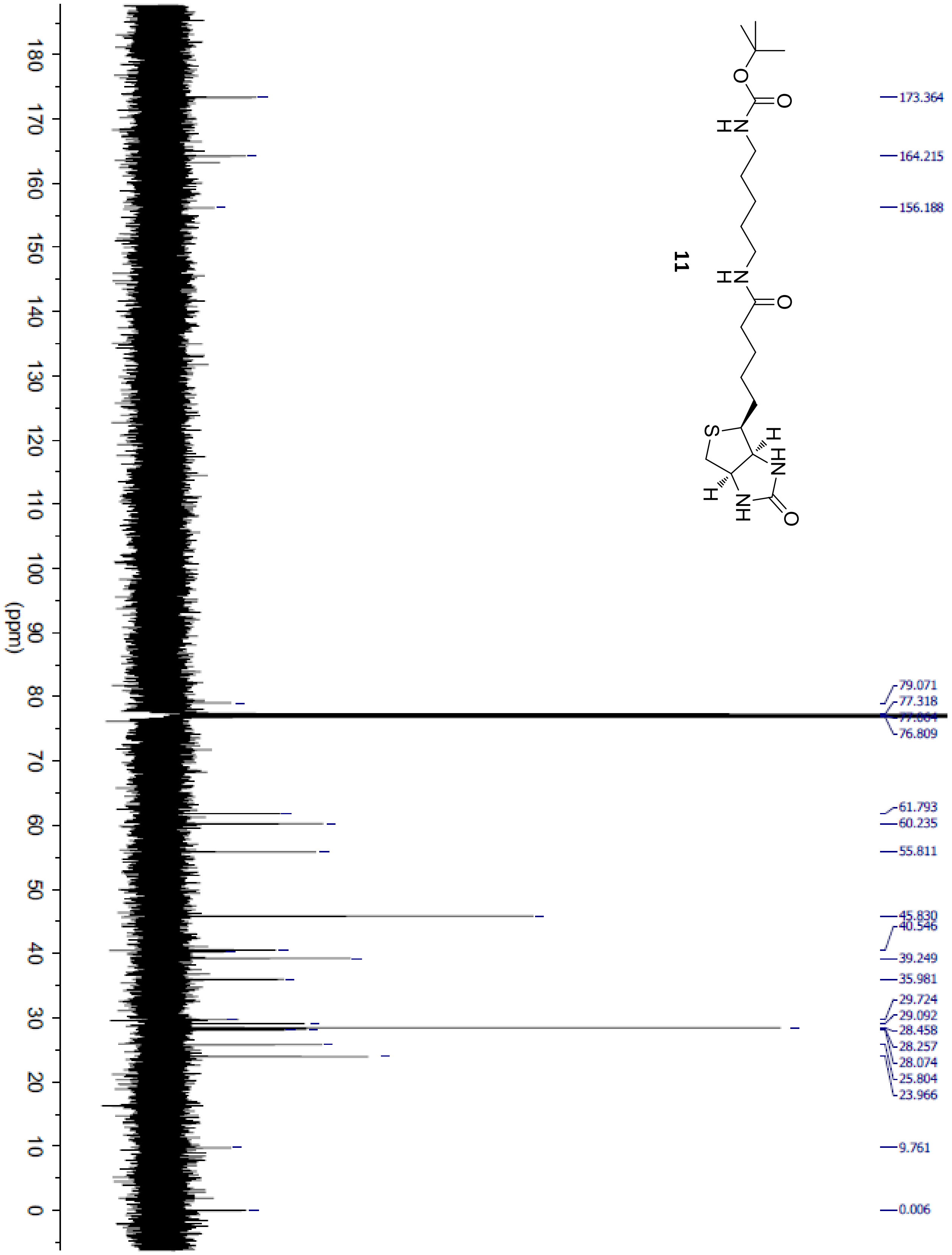

11
173.364
164.215
156.188
79.071
77.318
76.809
61.793
60.235
55.811
45.830
40.546
39.249
35.981
29.724
29.092
28.458
28.257
28.074
25.804
23.966
9.761
0.006
(ppm)
180
170
160
150
140
130
120
110
100
90
80
70
60
50
40
30
20
10
0

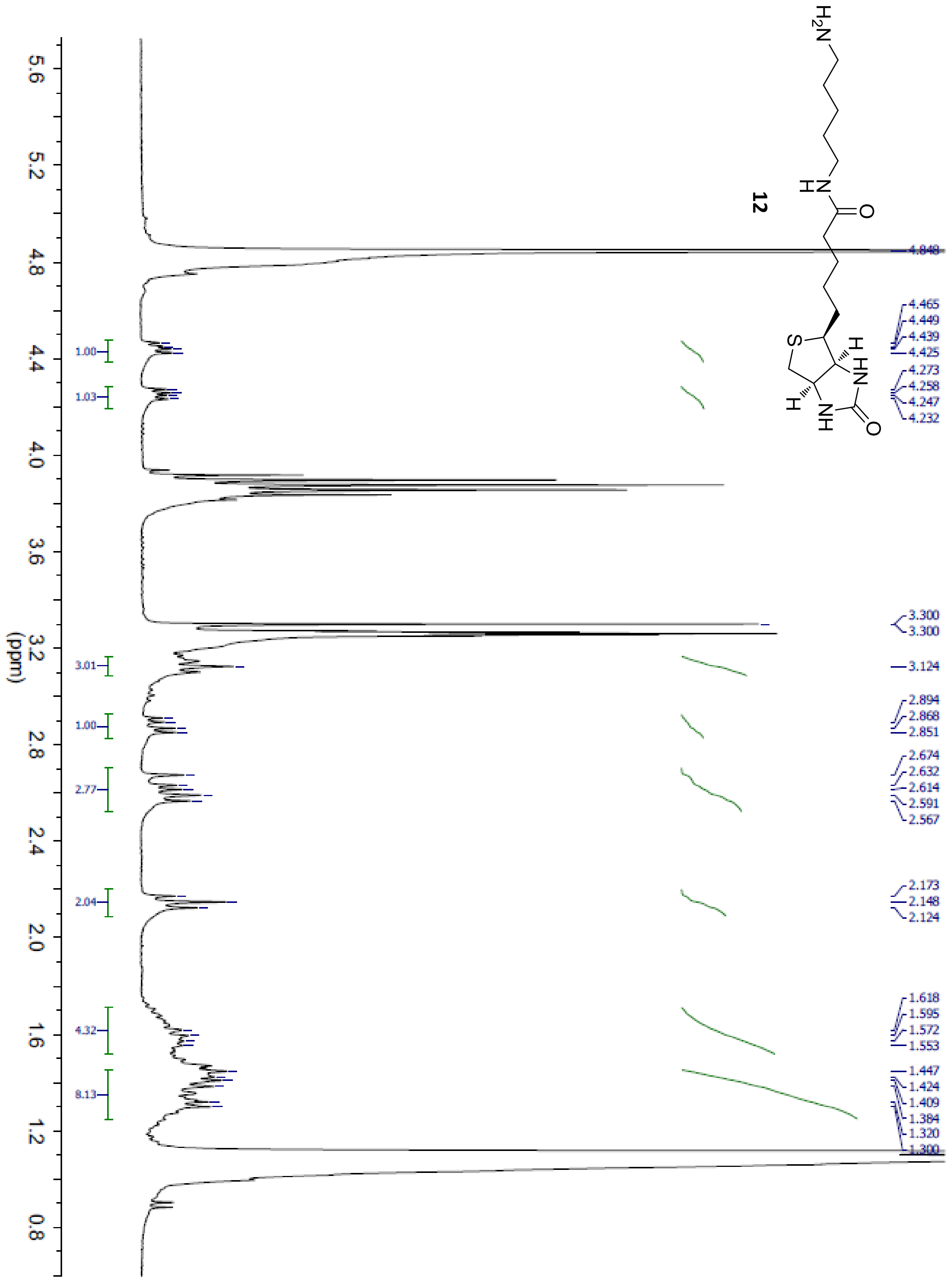

12
(ppm)
5.6
5.2
4.8
4.4
4.0
3.6
3.2
2.8
2.4
2.0
1.6
1.2
0.8
1.00
1.03
3.01
1.00
2.77
2.04
4.32
8.13
4.848
4.465
4.449
4.439
4.425
4.273
4.258
4.247
4.232
3.300
3.300
3.124
2.894
2.868
2.851
2.674
2.632
2.614
2.591
2.567
2.173
2.148
2.124
1.618
1.595
1.572
1.553
1.447
1.424
1.409
1.384
1.320
1.300
